# Spontaneous activity of the mitochondrial apoptosis pathway drives chromosomal defects, the appearance of micronuclei and cancer metastasis through the Caspase-Activated DNAse

**DOI:** 10.1038/s41419-022-04768-y

**Published:** 2022-04-07

**Authors:** Aladin Haimovici, Christoph Höfer, Mohamed Tarek Badr, Elham Bavafaye Haghighi, Tarek Amer, Melanie Boerries, Peter Bronsert, Ievgen Glavynskyi, Deborah Fanfone, Gabriel Ichim, Nico Thilmany, Arnim Weber, Tilman Brummer, Corinna Spohr, Rupert Öllinger, Klaus-Peter Janssen, Roland Rad, Georg Häcker

**Affiliations:** 1 Institute of Medical Microbiology and Hygiene, Medical Center, Faculty of Medicine, University of Freiburg, Freiburg, Germany; 2 Institute of Medical Bioinformatics and Systems Medicine, Medical Center –, Faculty of Medicine, University of Freiburg, Freiburg, Germany; 3 Comprehensive Cancer Center Freiburg, Medical Center, Faculty of Medicine, University of Freiburg, Freiburg, Germany; 4 German Cancer Consortium DKTK Partner Site Freiburg, German Cancer Research Center (DKFZ), Heidelberg, Germany; 5 Institute for Surgical Pathology, Medical Center, Faculty of Medicine, University of Freiburg, Freiburg, Germany; 6 Core Facility for Histopathology and Digital Pathology, Medical Center, Faculty of Medicine, University of Freiburg, Freiburg, Germany; 7 Cancer Research Center of Lyon (CRCL) INSERM 1052, CNRS 5286 Lyon, France; 8 Cancer Cell Death Laboratory, part of LabEx DEVweCAN, Université de Lyon, Lyon, France; 9 Institute of Molecular Medicine and Cell Research, Faculty of Medicine, University of Freiburg, Freiburg, Germany; 10 BIOSS Centre for Biological Signalling Studies, University of Freiburg, Freiburg, Germany; 11 Spemann Graduate School for Biology and Medicine, University of Freiburg, Freiburg, Germany; 12 Faculty of Biology, University of Freiburg, Freiburg, Germany; 13 Institute of Molecular Oncology and Functional Genomics, Department of Medicine II and TranslaTUM Cancer Center; TUM School of Medicine, Technical University of Munich, Munich, Germany; 14 School of Medicine, Dept. of Surgery, Technical University of Munich, 81675 Munich, Germany

**Keywords:** Metastasis, Apoptosis

## Abstract

Micronuclei are DNA-containing structures separate from the nucleus found in cancer cells. Micronuclei are recognized by the immune sensor axis cGAS/STING, driving cancer metastasis. The mitochondrial apoptosis apparatus can be experimentally triggered to a non-apoptotic level, and this can drive the appearance of micronuclei through the Caspase-activated DNAse (CAD). We tested whether spontaneously appearing micronuclei in cancer cells are linked to sub-lethal apoptotic signals. Inhibition of mitochondrial apoptosis or of CAD reduced the number of micronuclei in tumor cell lines as well as the number of chromosomal misalignments in tumor cells and intestinal organoids. Blockade of mitochondrial apoptosis or deletion of CAD reduced, while experimental activation CAD, STING-dependently, enhanced aggressive growth of tumor cells in vitro. Deletion of CAD from human cancer cells reduced metastasis in xenograft models. CAD-deficient cells displayed a substantially altered gene-expression profile, and a CAD-associated gene expression ‘signature’ strongly predicted survival in cancer patients. Thus, low-level activity in the mitochondrial apoptosis apparatus operates through CAD-dependent gene-induction and STING-activation and has substantial impact on metastasis in cancer.

## Introduction

During apoptosis, mitochondria release intermembrane proteins in a process known as mitochondrial outer membrane permeabilization (MOMP). These proteins activate cytosolic caspases and trigger apoptotic cell death [1]. It is becoming clear that it is experimentally possible to trigger the permeabilization of only a small share of a cell’s mitochondria, a process that has been termed minority MOMP [2]. In that study, using a Bcl-2-family inhibitor at sub-lethal doses, the authors demonstrated that the selective release of cytochrome *c* from these few mitochondria caused limited activation of caspases, which was insufficient to kill the cell. It was however sufficient to activate the Caspase-activated DNAse (CAD) by the caspase-dependent cleavage of its inhibitor ICAD [3, 4]; CAD-activity in the absence of apoptosis had earlier been identified during prolonged mitotic arrest [5] and in live, differentiating myoblasts [6]. There is now substantial evidence that components of the apoptosis signaling pathway also function outside cell death; this includes caspases as well as DNAses, and both CAD and the mitochondrial DNAse EndoG have been implicated [7]. Proposed functions of these signals mostly concern cell differentiation [7, 8]. One study reported that spontaneous activity of the effector caspases-3, −6 and −7 in the breast cancer cell line MDA-MB-231 was required for spontaneously appearing DNA-damage through CAD and EndoG. This pathway promoted aggressive growth of the cells in vitro, as well as growth of individual xenotransplanted tumors in mice, and it was proposed that the kinase ATM, a central component of DNA-damage signaling, mediated the biological effects [9].

Signaling originating from damaged DNA has been increasingly implicated in cell biology and immunology. One intriguing concept is the signal-generating capacity of micronuclei. Micronuclei arise in numerous situations of cell stress; although they are mostly detected in cancer cells [10], micronuclei can also be found in isolated epithelial cells from untreated murine colon [11]. When cells were subjected to repeated insults causing minority MOMP, an increased number of micronuclei have been found in the cells after a number of passages in cell culture [2], indicating that the limited activity of CAD in this experimental situation can contribute to micronuclei generation.

Micronuclei can arise from lagging chromosomes in the presence of mitotic spindle defects. They can also be generated from acentric chromosome fragments when cells with unrepaired DNA double-strand breaks go through mitosis [10]. This indicates the possibility that CAD-activity is immediately involved in the generation of mitochondria but this possibility has not been explored. Repeated CAD-activation during minority MOMP caused the appearance of micronuclei, and this phenotype has been hypothesized to be an expression of genomic instability induced by CAD [2]. A more recent study has found that melanoma cells that have recovered from the receipt of an apoptotic stimulus (‘failed apoptosis’) also exhibited more aggressive growth. This did not depend on continuing mitochondrial signals or on caspase-activity [12], suggesting that it was due to persistent mutations. A history of CAD-activity is thus linked to mutations and micronuclei.

High numbers of micronuclei are found in many cancers [10]. Intriguingly, an association of metastatic activity of cancer cells and micronuclei has been reported. This association has been ascribed to the cytosolic recognition of micronuclei by the innate immune receptor cyclic GMP-AMP synthase (cGAS): cGAS can be activated by micronuclei-DNA, to produce the stimulatory ligand for the signaling mediator, stimulator of interferon induced genes (STING). STING-signaling then drives a complex gene expression program, enhancing the metastatic activity of tumor cells [13].

We hypothesized that micronuclei, which are spontaneously generated in cancer cells, are to some extent the result of spontaneous minority MOMP and associated spontaneous CAD-activity. Apoptosis is very common in cancer cell lines in culture, and apoptotic cells can be detected during growth of numerous types of tumors in vivo. Because the signaling of minority MOMP appears to use the same machinery as apoptosis (i.e., MOMP), the near-ubiquitous occurrence of apoptosis suggests a similarly widespread prevalence of minority MOMP in cancer cells. Spontaneous caspase-dependent activation of CAD may therefore contribute to micronuclei generation. In this way, CAD may generate a ligand for cGAS/STING and drive metastasis.

## Results

### CAD contributes to the formation of micronuclei and chromosomal misalignment at steady state

**Fig. 1:**
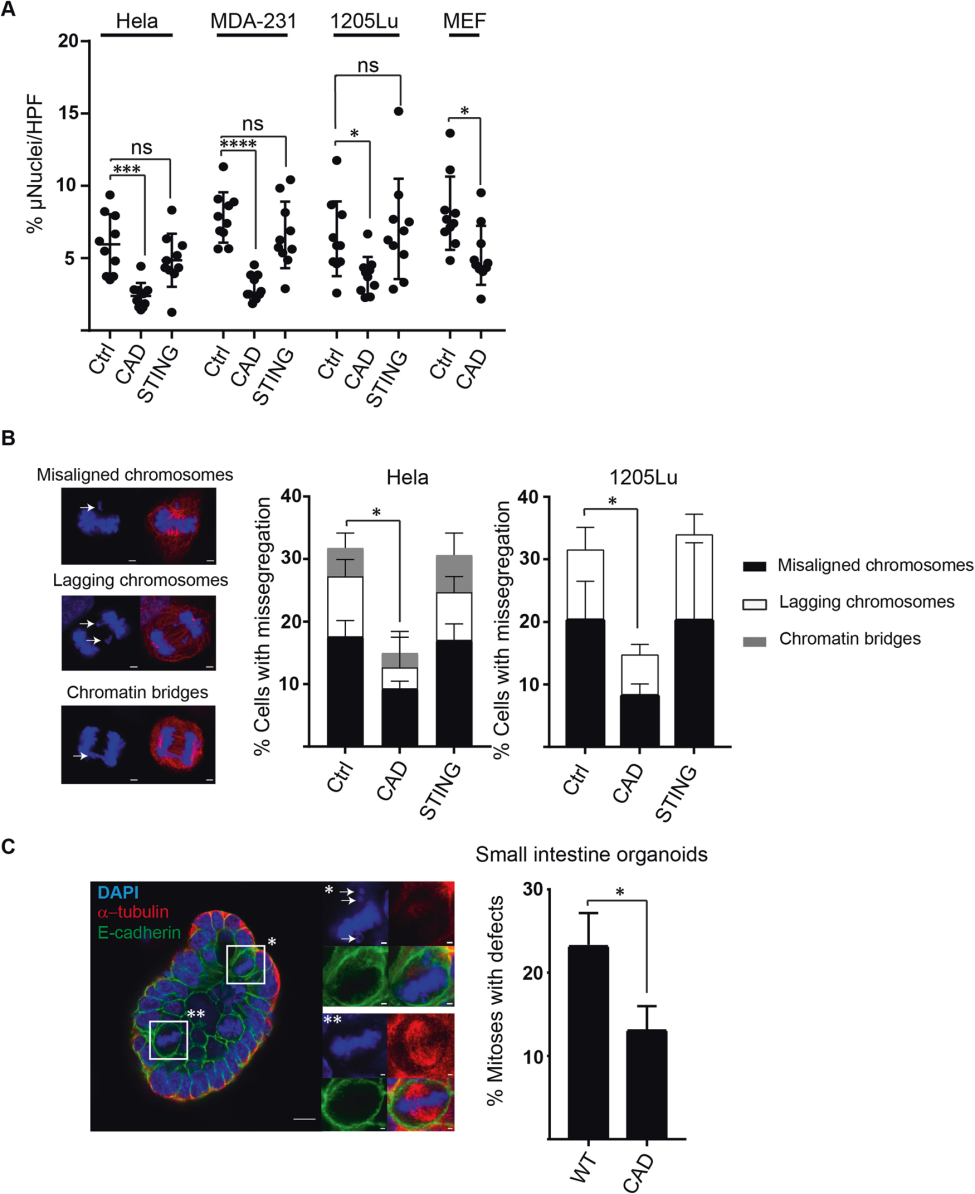
Spontaneous CAD-activity drives chromosomal missegregation and the appearance of micronuclei. **A** Percentage of micronuclei per cell. HeLa, MD-MB-231, 1205Lu and SV40-transformed mouse embryonic fibroblast (MEF) cells were stained with DAPI and beta-tubulin and assessed by microscopy for micronuclei content. CAD/STING are gene deficient mutants, Ctrl are control cells carrying a non-coding gRNA. MEFs were derived from wt or CAD-deficient mice. A minimum of 500 cells per genotype were counted. Symbols give the percentages of micronucleus-positive cells per high power field. Columns/error bars are mean/SD of 10 high power fields. *p < 0.05, ***p < 0.001, ****p < 0.0001. **B** Chromosome missegregation in Hela and 1205Lu cells. Representative images of misaligned and lagging chromosomes, as well as chromatin bridges in Hela cells (left) and quantification of chromosome missegregation (right). HeLa and 1205Lu cells (control cells carrying a non-coding gRNA or cells deficient in CAD or STING) were stained with DAPI (blue) and α-tubulin (red). A minimum of 100 anaphase/ telophase/ metaphase cells were counted and analyzed for mitotic defects. Data are means/SD from three independent experiments; *p < 0.05. **C** Mitotic defects in mouse small intestinal organoids. Organoids from wt or CAD-deficient mice were synchronized, stained with DAPI (blue), α-tubulin (red) and E-cadherin (green) and scored for metaphase cells. One hundred metaphase cells were counted and analyzed for mitotic defects. Insets show normal metaphase cells (**) or metaphase cells with defect (arrow) (*). Data shown are means/SD from three independent experiments; *p < 0.05.

We deleted CAD in three human tumor cell lines of different tissue origin, HeLa (cervical carcinoma), MDA-MB-231 (breast carcinoma) and 1205Lu (metastatic melanoma) (Fig. S1A). The deletions had no impact on cell viability or clonogenic survival (Fig. S1B). In standard conditions, all three cell lines showed the presence of micronuclei in about 5–10% of the cells (Fig. 1A, Fig. S2). Deletion of CAD reduced this number by about 50%. As a control, we deleted STING. STING can act as a downstream signaling mediator of micronuclei-recognition but should not affect the generation of micronuclei, and STING-deficient cell lines had similar numbers of micronuclei as the control cells (Fig. 1A). Mouse embryonic fibroblasts (MEFs) transformed with the SV40 large T antigen also contained significant numbers of micronuclei, and SV40-transformed MEFs from CAD-deficient mice also showed reduced numbers of micronuclei (Fig. 1A). Spontaneous activity of CAD thus contributes to the spontaneous generation of micronuclei.

Micronuclei often arise from chromosomal misalignments and mitotic defects. We observed a substantial number of defects in mitosis in control HeLa cells, such as misaligned or lagging chromosomes or chromosome bridges. The number of these defects was reduced by about half in CAD-deficient HeLa cells but unaltered by the deficiency in STING (Fig. 1B). Intriguingly, chromosomal misalignment is not confined to cancer cells but has been observed in non-transformed mouse intestinal organoids [14]. We established small intestine organoids from wt or CAD-deficient mice and found that CAD-loss again reduced the number of defective mitotic events by about half (Fig. 1C). The results show that CAD contributes to mitotic defects in proliferating epithelial cells, which likely is the origin of micronuclei.

### Spontaneous activity of the mitochondrial apoptosis system contributes to the generation of micronuclei

**Fig. 2:**
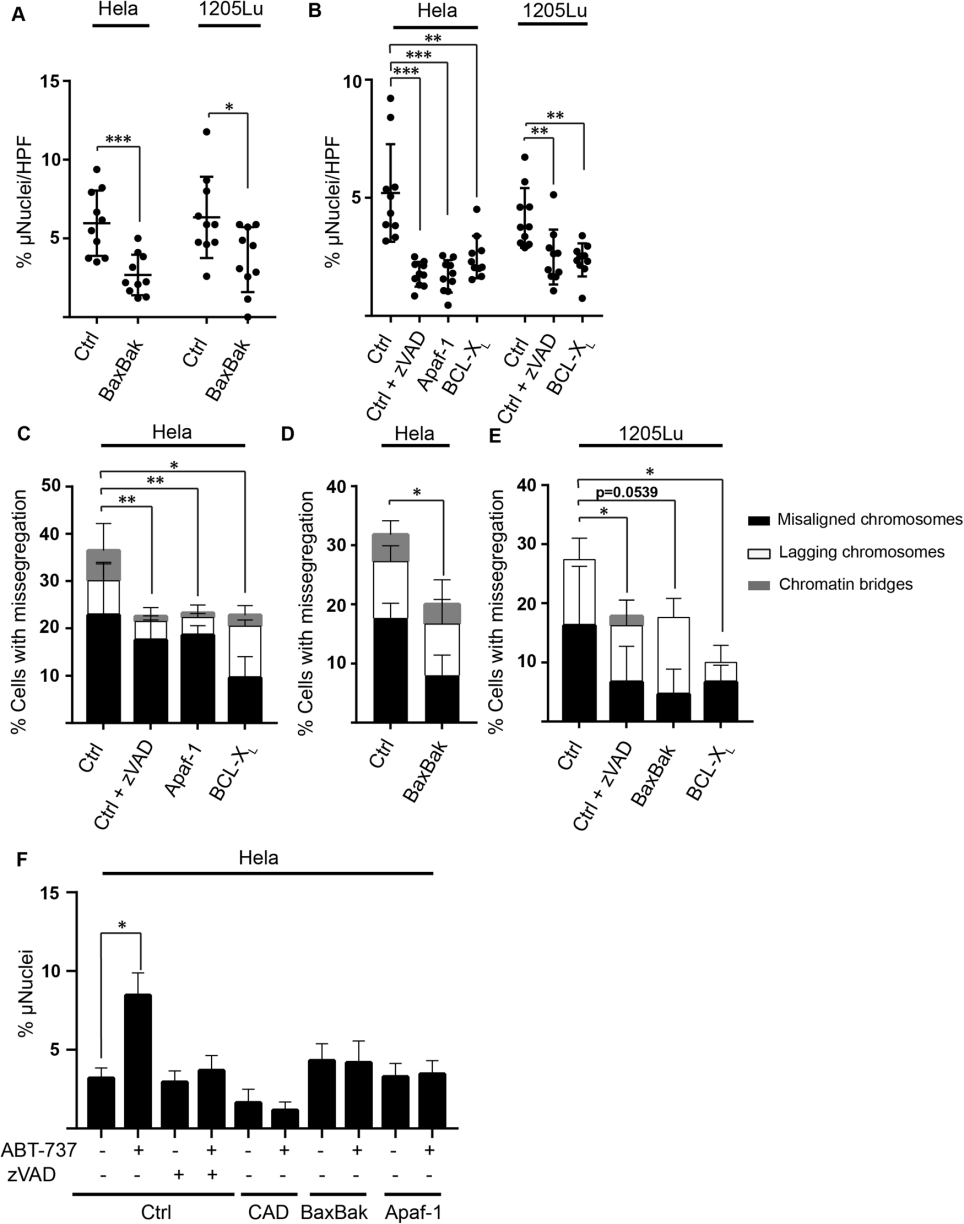
Spontaneous activity of the mitochondrial apoptosis system contributes to the generation of micronuclei. **A** Percentage of micronuclei per cell. HeLa and 1205Lu cells (control cells carrying a non-coding gRNA or cells deficient in both Bax and Bak) were stained with DAPI and beta-tubulin and assessed by microscopy for micronuclei. A minimum of 500 cells per genotype were counted. Symbols give the percentages of micronucleus-positive cells per high power field. Columns/error bars are mean/SD of 10 high power fields. *p < 0.05, ***p < 0.001. **B** Hela and 1205Lu cells (control cells carrying a non-coding gRNA, cells deficient in Apaf-1, cells overexpressing Bcl-X_L_ and cells treated with z-VAD (25 µM) for 24 h were stained with DAPI and beta-tubulin and assessed by microscopy for micronuclei content. A minimum of 500 cells per genotype were counted. Symbols give the percentages of micronucleus-positive cells per high power field. Columns/error bars are mean/SD of 10 high power fields. **p < 0.01, ***p < 0.001. **C**–**E** Chromosome missegregation in Hela and 1205Lu (control cells carrying a non-coding gRNA or cells deficient in Bax and Bak, cells deficient in Apaf-1, cells overexpressing Bcl-X_L_ and cells treated with z-VAD (25 µM), for 24 h). A minimum of 100 anaphase/ telophase/ metaphase cells were counted and analyzed for mitotic defects. Data shown are means/SD of three independent experiments. **p < 0.01 *p < 0.05. **F** Hela cells (control cells carrying a non-coding gRNA, cells deficient in Apaf-1 or Bax and Bak and cells treated with z-VAD (25 µM) were treated with ABT-737 (10 µM) for 24 h and analyzed for micronuclei by flow cytometry. Data represent the mean/SD of three independent experiments. *p < 0.05.

The only known way to CAD-activation is by the caspase-dependent cleavage of its inhibitor. This suggests that the CAD-dependent chromosomal misalignments and generation of micronuclei is the result of continuous, sub-lethal caspase-activity, which may arise as a consequence of low-level activity of the mitochondrial apoptosis apparatus (minority MOMP) as reported previously [7, 9]. We tested this by targeting components of the mitochondrial apoptosis system. Deletion of Bax and Bak in HeLa and in 1205Lu cells reduced the number of micronuclei at steady state (Fig. 2A). A similar effect was seen when Bcl-X_L_ was over-expressed, when the cytosolic activator of caspases Apaf-1 was deleted or when caspases were inhibited (Fig. 2B). All approaches to block apoptosis-signaling also significantly reduced the number of cells with detectable chromosomal missegregation (Fig. 2C–E). These data show that spontaneous activity of the mitochondrial apoptosis apparatus drives chromosomal missegregation and the appearance of micronuclei.

The induction of minority MOMP with ABT-737 increased the number of micronuclei after 5–10 passages. We found that single treatment with ABT-737 over 24 h was sufficient to cause the appearance of increased numbers of micronuclei, and this was not seen in the absence of CAD, Bax/Bak or Apaf-1 or in the presence of a caspase-inhibitor (Fig. 2F). Taken together, these data confirm that the mitochondrial apoptosis pathway is spontaneously active in tumour cells in cell culture, which through the caspase-dependent activation of CAD causes the generation of micronuclei. They further show that it is continuous activity in this pathway rather than serial activation and persistent downstream effects that is required for chromosomal missegregation and the generation of micronuclei.

### The mitochondrial apoptosis pathway and CAD determine growth behavior of tumor cells in vitro

**Fig. 3:**
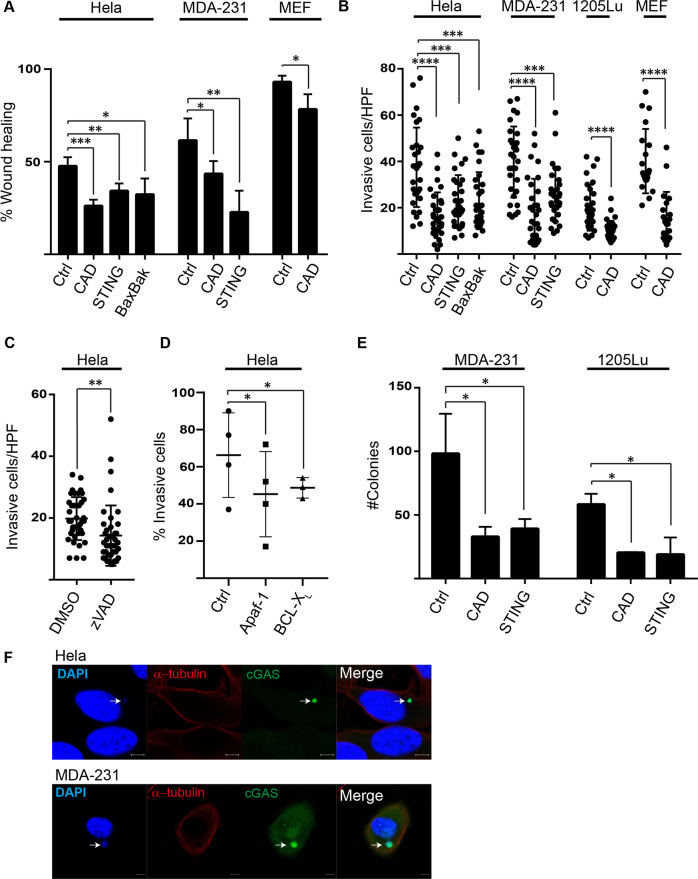
The mitochondrial apoptosis pathway and CAD determine growth behavior of tumor cells in vitro. **A** Deficiency in CAD, STING and Bax and Bak reduce migration. Hela, MDA-MB-231 and MEF cells (control cells carrying a non-coding gRNA or cells deficient in CAD, STING and Bax and Bak) were subjected to scratch assay, and wound closure was recorded. Data shown are mean/SD of three independent experiments. *p < 0.05, **p < 0.01, ***p < 0.001. **B** Hela, MDA-MB-231, 1205Lu and SV40-transformed MEF cells (control cells carrying a non-coding gRNA or cells deficient in CAD, STING and Bax and Bak; MEFs from wt or CAD-deficient mice) were tested for invasion through an ECM basement membrane in cell culture. The number of cells that had migrated after 24 h is shown. Ten high power fields were counted in each experiment, represented by individual symbols. Data shown are means/SD of three independent experiments. ***p < 0.001, ****p < 0.0001. **C** Hela cells (treated with 10 µM zVAD for 24 h) were tested for invasion through an ECM basement membrane in cell culture. The number of cells that had migrated after 24 h is shown. Ten high power fields were counted in each experiment, represented by individual symbols. Data shown are means/SD of three independent experiments. **p < 0.01. **D** Hela cells (control cells carrying a non-coding gRNA or cells deficient in Apaf-1 or cells overexpressing Bcl-X_L_) were tested for invasion through a Matrigel basement matrix. The percentage of cells that had migrated after 24 h is shown. Data shown are means/SD of three independent experiments. *p < 0.05. **E** Anchorage-independent growth of MDA-MB-231 breast cancer and 1205Lu melanoma cells (control cells carrying a non-coding gRNA or cells deficient in CAD or STING) was measured as the number of colonies formed in soft-agar. Colonies were stained and counted after 21 days. Data shown are means/SD of three independent experiments. *p < 0.05. **F** cGAS localization to micronuclei. Hela and MDA-MB-231 cells were fixed and stained for cGAS (green). DAPI (blue) and α-tubulin (red) were used for the staining of nucleus/micronucleus and cytoskeleton respectively. Arrows show micronuclei. Images are representative of at least 10 pictures per cell lines. Scale bar: 5 µM.

The presence of micronuclei is associated with STING-dependent invasive growth of cancer cells in vitro [13]. The spontaneous activity of the mitochondrial apoptosis pathway has been shown to be linked to invasion in MDA-MB231 cells [9]. We confirmed and extended this finding: CAD-deficient tumor cells showed reduced migration and wound healing by scratch assay, and the same was the case for SV40-transformed MEFs from CAD-deficient animals (Fig. 3A and S3A, B). Invasive growth was likewise reduced in cells lacking CAD (Fig. 3B), as was the formation of colonies in soft agar (Fig. 3E). The deletion of Bax/Bak or Apaf-1, the overexpression of BCL- X_L_ or the treatment with caspase-inhibitor in HeLa cells had a very similar effect (Fig. 3C, D). In all three assays, the behavior of CAD-deficient cells was comparable to the one of STING-deficient cells (Fig. 3A–C). Staining for endogenous cGAS, we obtained a strong signal in micronuclei (Fig. 3F), confirming that cGAS may be activated in micronuclei generated through CAD-activity [15, 16]. In HeLa cells, we observed phosphorylated STING by Western blotting in control cells, which was strongly reduced in CAD-deficient cells; mRNA-expression of IFNα and IFNβ was detected in control HeLa cells, and this was significantly reduced in CAD- or STING-deficient cells (Fig. S3C). No type I mRNA was detectable in MDA-MB231 control cells (not shown). These results expand the observations that spontaneous activity in the pathway from mitochondrial apoptosis-regulation, over caspase-activity to CAD-activation determines growth behavior. Deletion of STING gave a very similar result in these assays to blockade of the mitochondria-CAD-signaling pathway. This is consistent with the hypothesis that CAD-dependent micronuclei are the substrate for the STING signaling axis.

### Experimental activation of CAD can drive STING-dependent invasion in vitro

The data indicated that an axis from mitochondria to CAD contributed to invasive growth of tumor cells. We next asked whether CAD-activity on its own could drive invasion in cell culture. Because the cell lines tested so far are very invasive, we chose a less aggressive cell line for these experiments, HaCaT human keratinocytes. HaCaT cells are a line of spontaneously immortalized keratinocytes with normal differentiation potential [17]. We established a system for targeted destruction of the CAD-inhibitor ICAD by fusion to a small protein tag, an ‘auxin-induced degron’ (AID) [18]. When the plant F-box protein Tir1 is co-expressed, the addition of the plant hormone auxin induces the degradation of ICAD, leading to the activation of CAD; a similar system has been reported in a chicken cell line [19], and we adopted this system with modifications.

**Fig. 4:**
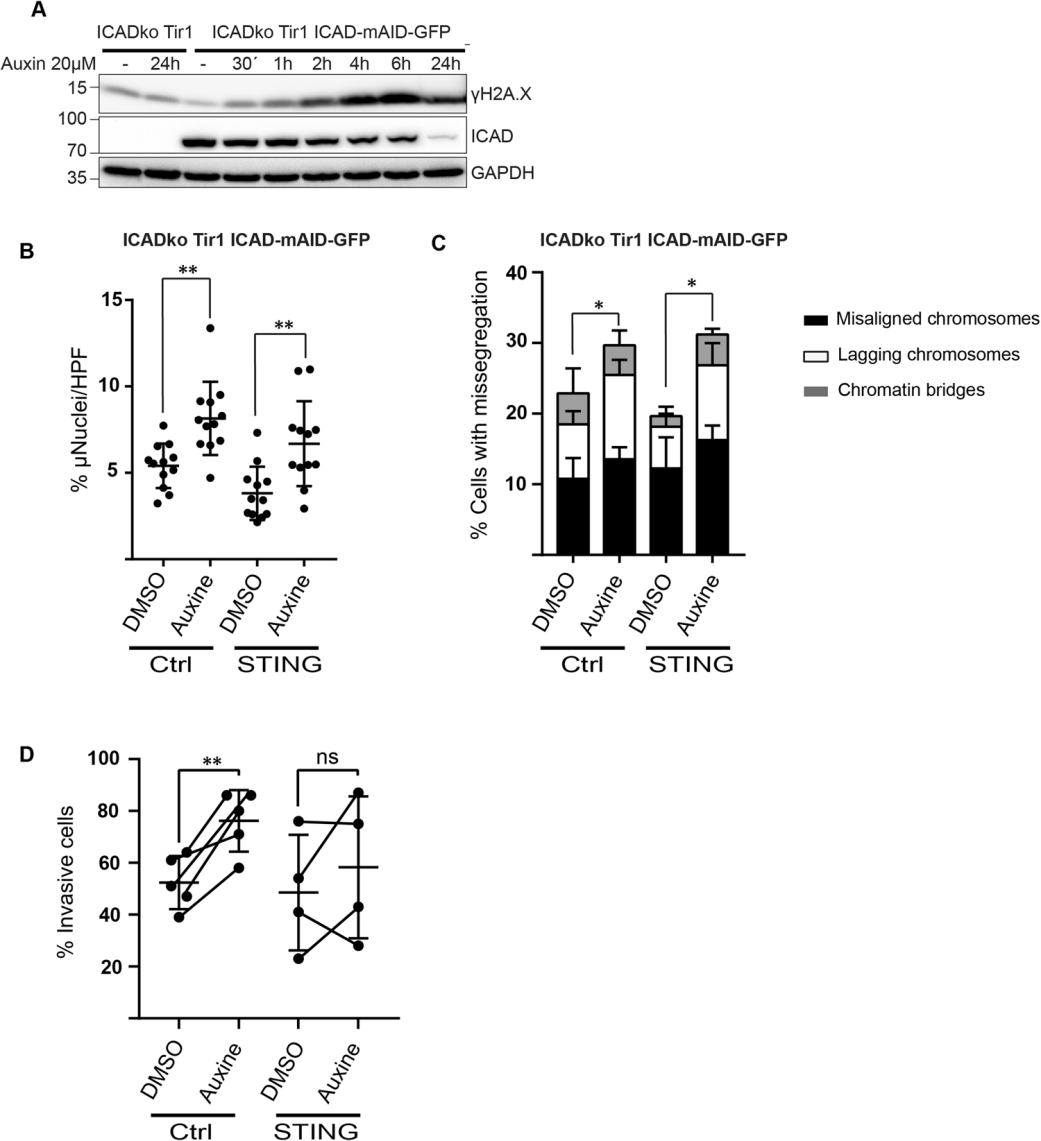
Experimental activation of CAD can drive STING-dependent invasion in vitro. **A** HaCaT cells with a deletion in endogenous ICAD were engineered to express an AID-ICAD-GFP construct. Cells were treated with auxin (20 µM) over 24 h. Western blot shows loss of ICAD and the appearance of a DNA-damage response (γH2AX) over time. **B** HaCaT AID-ICAD-GFP cells (see **A**) were further modified by the CRSIPR/Cas9-mediated deletion of STING. Cells were treated with solvent (DMSO) of auxin (20 µM) for 6 h. Auxin was washed out, and culture was continued for 24 h. Cells were stained with DAPI and beta-tubulin and assessed by microscopy for micronuclei. A minimum of 10,000 cells per group were counted. Symbols give the percentages of micronucleus-positive cells per high power field. Columns/error bars are mean/SD of 10 high power fields. **p < 0.01. **C** Chromosome missegregation in HaCaT AID-ICAD-GFP cells (control or additional deficient in STING as in **B**). A minimum of 500 anaphase/ telophase/ metaphase cells were counted and analyzed for mitotic defects. Data shown are means/SD of three independent experiments. *p* < 0.05. **D** HaCaT AID-ICAD-GFP cells (control or additional deficient in STING as in **B**) were treated with auxin (20 µM) and tested for invasion through a Matrigel basement matrix. The percentage of cells that had migrated after 24 h is shown. Data shown are means/SD of 4–5 independent experiments. **p < 0.01.

We engineered HaCaT cells where endogenous ICAD had been replaced with AID-ICAD. Auxin-treatment caused the loss of ICAD, paralleled by the appearance of the DNA-damage response marker γH2AX indicative of CAD-activation (Fig. 4A). The activation of CAD increased the number of micronuclei and chromosomal missegregations as expected, and the deletion of STING (Fig. S1C) had no effect on this (Fig. 4B, C). We used this gain-of-function model to test whether the activation of CAD could enhance invasion in HaCaT cells. Indeed, direct activation of CAD by auxin-mediated ICAD-degradation enhanced invasion of HaCaT cells (Fig. 4D), indicating that in these cells CAD-activity is sufficient to increase the potential of the cells for invasive growth. In STING-deficient HaCaT cells, however, no enhanced invasion was observed upon activation of CAD (Fig. 4D). Our results show that spontaneous activity of CAD causes the appearance of micronuclei in human cells, correlating with STING-dependent aggressive growth behavior. As discussed above, it has been reported that metastasis of MDA-MB231 breast cancer cells in mice was reduced when STING had been silenced [13]. This suggests that CAD generates micronuclei as a ligand for cGAS, whose activity then drives the activation of STING and subsequently a metastasis-enhancing gene expression profile.

### CAD is a determinant of metastatic potential in vivo

**Fig. 5:**
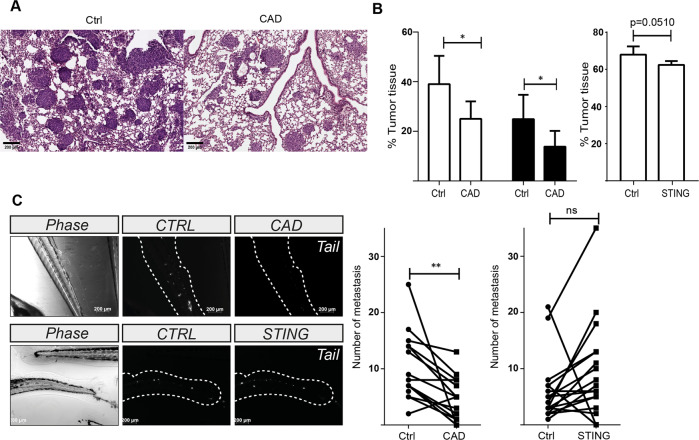
CAD is a determinant of metastatic potential in vivo. **A** Formation of melanoma metastases in mice. 1205Lu human melanoma cells (carrying a non-coding gRNA (CTRL) or deficient in CAD) were injected in the tail vein of Rag2^−/−^γ(c)^−/−^ mice (6 mice per group). Mice were sacrificed after 15 days, and the percentage of tumor tissue was assessed in H&E stained lung sections. One exemplary picture for each genotype is shown. **B** Percentage of tumor tissue in lungs of mice injected with melanoma cells (CTRL, CAD and STING deficient cells). Manual (white bars) and digital (black bars) quantifications are shown. Injections of control and CAD- or STING-deficient cells to groups of mice were done in parallel. Data shown are means/SD of six mice each. *p < 0.05. **C** Formation of metastases in zebrafish. Zebrafish embryos were injected with fluorescently labelled MDA-MB-231 cells (carrying a non-coding gRNA (CTRL) or deficient in CAD or STING; about 300 cells per embryo). In each experiment one embryo was injected with control (CTRL) and one with either CAD- or STING-deficient cells. After 48 h, metastases that had formed in the caudal region of the embryos were counted. Pictures show representative experiments for injection of CAD- (top) or STING- (bottom) deficient cells.

Spontaneous activity of caspases and CAD has been reported to drive growth rates of subcutaneously xenotransplanted tumors in mice [9], while STING has been found to regulate metastasis [13]. Our results suggested that CAD-activity contributes to metastasis of cancer cells through the activation of STING-signaling. We xenografted control or CAD-deficient 1205Lu human melanoma cells intravenously into immunocompromised mice and measured the development of lung metastases. CAD-deficient 1205Lu cells showed a significant reduction in the generation of lung metastases (Fig. 5A, B). No significant effect on metastasis formation was observed for STING-deficiency (Fig. 5B). As a second in vivo-model, we used zebrafish larvae. Zebrafish larvae have emerged as a suitable system to model the metastatic potential of human cancer cells [20]. We injected MDA-MB-231 control or CAD-deficient cells into the perivitelline cavity of the larvae and tested for the invasion of cancer cells into the caudal blood vessels. CAD-deficient cells showed significantly reduced potential to form metastases (Fig. 5C). Similar to the mouse xenograft results, there was no reduction of metastatic behavior by STING-deficiency (Fig. 5C). These results support the phenotype observed in vitro and suggest that CAD-activity is an important determinant of invasion and metastasis formation in human cancer cells.

### CAD drives a cell-specific gene expression profile

**Fig. 6:**
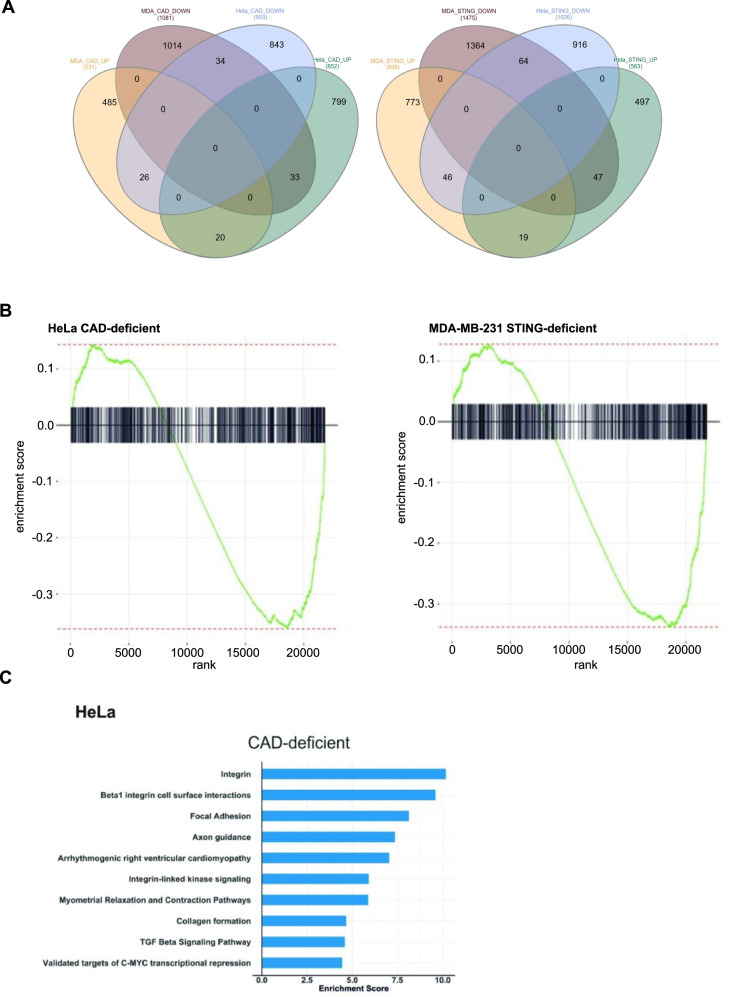
CAD drives a cell-specific gene expression profile. **A** Venn-diagrams comparing expression profiles of CAD- and STING-deficient Hela or MDA-MB-231 cells. The numbers of differentially expressed genes are shown. **B** Enrichment analysis of EMT-related genes in Hela CAD-deficient (left) and MDA-MB-231 STING-deficient (right) cell lines. **C** Functional enrichment analysis using Fisher’s exact test. The top-10 negatively enriched consensus terms are shown in the bar plots. The enrichment score presents the corresponding log10 *p* value.

The results suggested that CAD-activity changes the gene-expression profile of the cell in a way that affects growth behavior. To determine this profile, we performed RNA sequencing on two sets of cell lines, MDA-MB-231 and HeLa, in both cases of control and of cells that were either CAD- or STING-deficient. Principal Component Analysis (PCA) showed distinct clustering of the genotypes (Fig. S4). For both cell lines and each genotype, a large set of differentially expressed genes (DEG) was identified. There was substantial overlap between genes deregulated in CAD-deficient and STING-deficient cells for each cell line, consistent with the interpretation that both molecules regulate overlapping but non-identical signaling pathways. Surprisingly, the overlap between the genes deregulated in the two different cell lines of the same genotype was much smaller: 54 genes were identified as DEG in both HeLa and MDA-MB231 cells in CAD-deficiency, and 83 DEG were consistently found in STING-deficiency (Fig. 6A).

In MDA-MB-231 cells, micronuclei have been found to correlate with an up-regulation of genes involved in epithelial-mesenchymal transition (EMT), a trait that links up to invasive behavior and metastasis [13]. STING-deficient MDA-MB231 although not HeLa cells showed downregulation of EMT-genes (Fig. 6B, Fig. S5A). Conversely, CAD-deficiency was associated with the downregulation of EMT-genes in HeLa (Fig. 6B) but not in MDA-MB-231 cells (Fig. S5A). Pathway analysis in CAD-deficient HeLa cells showed deregulation of invasion-associated gene groups such as integrin and focal adhesion kinase signaling, as well as other cancer-related pathways (TGF-β- and myc-signaling) (Fig. 6C). Although CAD-deficient MDA-MB231 cells also showed deregulation of a set of pathways that may contribute to growth behavior (senescence/autophagy, nucleotide metabolism, transcription factor networks), the pathways were different from the ones identified in HeLa cells (Fig. S5B). A heatmap of the expression of the genes most significantly regulated by CAD/CAD-deficiency is shown in Fig. S6 for each cell line. Both CAD and STING therefore appear to regulate similar gene expression pathways, but this regulation to a substantial degree depends on cell line-specific factors.

### Expression of CAD-regulated genes is associated with poor prognosis in cancer patients

We established gene-expression signatures using genes that were down-regulated in CAD-deficient cell lines, on the assumption that CAD was required to maintain the expression of these genes (strictly speaking, this may not be an immediate CAD-signature, as other factors are likely to play a role, especially given the differences between the two cell lines. We will here use this term for want of a better one and will discuss it further below). We evaluated the relevance of these signatures for survival of cancer patients. For these signatures, down regulated genes (adjusted *p* < 0.01) in CAD deficient either HeLa or MDA-MB-231 cell lines were selected (Table S1, 2).

**Fig. 7:**
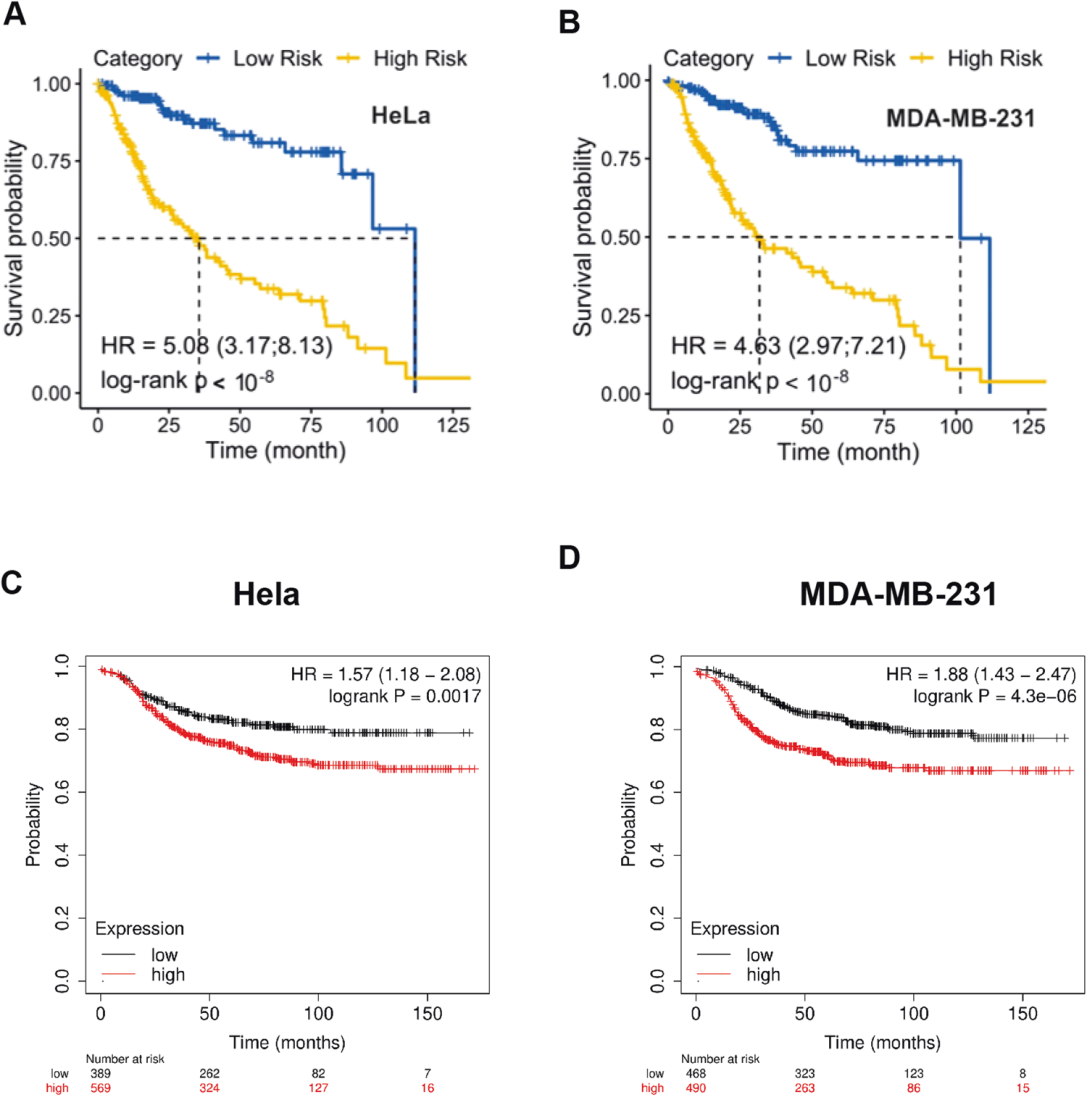
Expression of CAD-regulated genes is associated with poor prognosis in cancer patients. The prognostic impact of CAD deficient gene signatures on survival of patients from the TCGA data collection was examined using the CoxPH model. Association of a CAD-signature derived from Hela (**A**) and MDA-MB-231 (**B**) with survival. Signatures comprised of genes differentially expressed between control and CAD-deficient cells (padj < 0.01). Patients were assigned to high- vs. low-risk groups based on their expression levels of the signature genes in tumour vs. normal tissue. In all cases, a significant separation (log-rank *p* < 10^−8^) between high and low risk subjects is achieved. **C**, **D** Distant metastasis-free survival in a cohort of 958 breast cancer patients stratified according to the expression of the 10 most downregulated genes in each cell line.

We tested the association with these signatures with survival using gene expression data from public data bases. We first used a dataset from the cancer genome atlas (TCGA) by applying a multivariate Cox proportional hazards model (CoxPH model). Both signatures stratified patients to low/ high risk groups with highly significant *p* values (<10^−8^) (Fig. 7A, B): CAD-dependent gene regulation was associated with poor outcome. Because at least the majority of cancer patients die due to metastases [21] this is consistent with a role of CAD in driving metastasis. We further analyzed the expression levels of the signatures in established tumors and assessed their prognostic value. A highly significant correlation was found between the high expression of the signatures and poor prognosis for patients from another recently published study of breast cancer patients (Fig. 7C, D).

This uniformity was surprising and suggested that a number of different biological mechanisms, rather than one particular pathway, are capable of regulating growth behavior and, presumably through this, cancer patient survival. We therefore tested individual genes found in either cell line signature for their independent association with overall survival of patients in the relatively large study of gastric cancer patients. A set of 19 genes was analyzed in this way, namely the 10 genes most significantly deregulated by CAD-deficiency in either HeLa or MDA-MB231 cells (one gene was found in both datasets, and most genes were also deregulated in STING-deficient cells; Table S3). Twelve out of these 19 genes showed independent, individual association with survival in this patient cohort: patients with high expression of any of these genes had a less favorable prognosis than patients with low expression levels in their tumors (Fig. S7). CAD-dependent gene-regulation therefore shows a strong association with disease outcome for cancer patients, and CAD appears to exert this regulation through a number of gene products strongly associated with cancer progression. Because metastasis is a factor strongly predicting poor outcome, the most straightforward interpretation of these results is that the apoptotic pathway trough CAD-activation regulates genes that in turn drive invasion and metastasis of cancer cells.

## Discussion

We here extend on the association of the sub-lethal activity of the mitochondrial apoptosis apparatus and growth behavior of human cells. Spontaneous activity of the apoptotic signaling pathway could drive CAD-dependent aggressive growth of human cells, associated with metastasis in vivo. CAD-activity drove a gene expression program with input from STING, suggesting that one activity of CAD is the generation of a STING-ligand, presumably micronuclei.

The description of minority MOMP has illustrated the experimental possibility to induce a low-level, non-lethal activation of the pathway, providing the basis for a number of previous observations [7], and evidence has been presented that cancer cells harbor spontaneous activity in the mitochondrial apoptosis pathway [9]. Mitochondria play a role in the activation of the cell, for instance through the release of mitochondrial DNA/RNA [22–24], and they can determine cell behavior through their metabolism [25]. Our data confirm that there is at steady state, at least in cancer cells, a low-level leakage of mitochondrial pro-apoptotic factors. It most likely involves the same signals and protein components as during apoptosis. Mitochondria are rarely present as isolated individual organelles, and continuous fission and fusion generates a flexible network of mitochondrial structures. It is easily conceivable that these processes involve a certain leakage of intermembrane components, which may generate the activity we found.

The cellular reaction to these signals that we observe ties in with various levels of a DNA-damage response (DDR). STING-dependent signaling upon recognition of micronuclei appears to play a role, as well as additional elements that are presumably also part of the DDR. Although molecularly only in parts understood, DNA-damage through chemicals like etoposide [26], or through UV-irradiation [27], involves complex transcriptional responses and signaling. Notably, DNA-signaling has previously been linked to mitochondrial apoptosis [22, 23] and even sub-lethal signals [28]: mitochondrial DNA can be released into the cytosol during apoptosis [29, 30], where it may be recognized by cGAS. However, because CAD-signaling can trigger at least some STING-dependent signals and CAD is activated by mitochondrial signals, this pathway may also affect the cellular response.

STING was required for CAD-driven invasion in HaCaT cells, and the deletion of CAD or STING generated overlapping but non-identical gene expression-profiles in two tumor cell lines. STING therefore receives input from other sources, possibly mitochondrial DNA. CAD-dependent signals were partly STING-independent. DNA-damage responses, for instance through ATM [9] or ATR [31] kinases, may also contribute to the cell’s response to CAD-activity. A case in point is the transcriptional regulator KAP1/TRIM28, which is typically activated during a DDR (and which is rapidly phosphorylated upon CAD-activation in HaCaT AID-ICAD-GFP-cells, not shown). Targeting KAP in breast carcinoma cells reduced metastasis [32]. This and perhaps other STING-independent effects of the DDR may contribute to the pro-metastatic activity of CAD and may explain the more pronounced effect of CAD deletion vs. STING-deletion in out xenograft models.

A set of presumably CAD-regulated genes was associated with cancer progression in several cohorts. The term CAD-signature may not be appropriate because deregulation of these genes is not necessarily the direct result of CAD-activity. Indeed, the ‘signatures’ obtained from HeLa vs. MDA-MB231 cells were rather different, indicating that additional, cell-specific factors are involved in the regulation of these genes. For instance, the signatures may be an expression of genomic instability promoted by CAD. Nevertheless, CAD-activity can drive the regulation of a set of genes whose expression can predict disease-outcome.

Apoptosis is regarded as an anti-cancer mechanism, and the resistance to apoptotic cell death is even considered a hallmark of cancer [33]. Blockade of apoptosis can be a strong factor contributing to the malignant transformation of mammalian cells. It was therefore surprising to find an activity of the apoptotic signaling pathway that has the potential to drive the progression of established tumors. At the same time, there are numerous reports that are at odds with the pure view of apoptosis as an anti-cancer mechanism, but consistent with the effect we describe. Many studies have found that high expression levels of the anti-apoptotic protein Bcl-2 are associated with good rather than poor prognosis (reviewed in [34]). Low expression levels of the pro-apoptotic proteins Bax, Bak and Smac were highly significantly associated with prolonged progression-free survival in melanoma patients [35], and similar results were reported for the expression of pro-apoptotic Bik in breast carcinoma [36]. A low propensity to undergo apoptosis is therefore associated with a favorable prognosis once the tumor has been established, which seems incompatible with the anti-tumor activity of apoptosis. However, all available evidence indicates that apoptosis and sub-lethal apoptosis signaling use the same machinery [2, 28]. Little pro-apoptotic activity, such as in situations of high Bcl-2-expression, therefore very likely also means less sub-lethal apoptosis signaling and less CAD-activation. Our findings show that sub-lethal engagement of the mitochondrial apoptosis apparatus can drive aggressive growth, as shown before [9], but also metastasis. Because metastasis is a major determinant of cancer-development, the results suggest that the observed associations should be interpreted as a link of prognosis with sub-lethal mitochondrial signals and metastasis, rather than with full apoptosis.

If sub-lethal apoptosis signals can worsen the prognosis of cancer, this has important implications for cancer therapy. Most traditional adjuvant cancer therapies, such as irradiation and chemotherapy, but also newer targeted therapies, for instance Bcl-2-family inhibition, aim at inducing apoptosis. Some tumor cells will escape apoptosis but may activate signaling to a low level, which may be unfavorable for the patient. The apoptosis apparatus, and in particular CAD itself, may be attractive drug targets for the therapeutic inhibition of cancer progression.

## Materials and methods

### Cell lines and culture conditions

HeLa229 (HeLa) cells (ATCC Cat# CCL-2.1) and MDA-MB-231 cells (ATCC Cat# HTB-26) were cultured in RPMI 1640 medium (Thermo Fisher Scientific, Gibco) with 10% FCS (Sigma–Aldrich, #F7524). Mouse embryonic fibroblast (MEF) cells transformed with SV40 large T-antigen were generated from wild-type and CAD KO embryos and were cultured in DMEM (Thermo Fisher Scientific) with 10% FCS and 50 µM 2-mercaptoethanol. The metastatic melanoma cell lines 1205Lu (Dr Meenhard Herlyn, Wistar Institute, Philadelphia) were cultured in TU2% melanoma medium containing 80% (v/v) MCDB153 (Sigma–Aldrich, #M7403), 20% (v/v) Leibovitz’s L-15, 2% (v/v) FCS (Thermo Fisher, Gibco), 5 µg/ml insulin (bovine, Sigma–Aldrich, #I4011), and 1.68 mM CaCl_2_. 293FT cells (Invitrogen) were cultured in DMEM/10%FCS medium. HaCaT keratinocytes [17] were cultured in DMEM supplemented with 10% FCS (Anprotec) and 1% (v/v) penicillin/streptomycin (Gibco). Auxin (IAA, 3-indoleacetic acid, Sigma, I2886) was dissolved in DMSO as 50 mM stock solution and used as indicated. All cells were cultured at 37 °C with 5% CO_2_. Gene-deficient cells were generated by CRISPR/Cas9 genome editing by transducing the cells with the lentiviral vector lentiCRISPR v2 (Addgene; Sanjana et al., 2014) and selection with puromycin (Invivogen). Guide RNAs were CTRL (non-targeting control) (ATCGTTTCCGCTTAACGGCG) or targeting CAD (TCGGCGTTGTCGGGAACACT) or STING (GCTGGGA CTGCTGTTAAACG). Cell lines were tested negative for mycoplasma contamination using two different kits: Venor^®^GeM Mycoplasma Detection Kit for conventional PCR version 1.4 and Lonza MycoAlert™ mycoplasma detection kit.

### Antibodies

Primary antibodies used were anti-γH2AX (#2577, Cell Signaling), anti-GAPDH (MAB374, Millipore), anti-CAD (PA5-19913, Thermo Fisher), anti-STING (#13647, Cell Signaling), anti-phospho-STING (#19781, Cell Signaling), anti-ICAD (#9732, Cell Signaling), anti-cGAS (#79978, Cell Signaling), anti-E-cadherin (#3195, Cell Signaling) and anti-α-tubulin(T9026, Sigma–Aldrich). Secondary antibodies were (Western blotting) anti-rabbit IgG(H + L)-HRP (A6667, Sigma-Aldrich), anti-mouse IgG(H + L)-HRP (115-035-166, Dianova), (immunofluorescence) anti-mouse IgG(H + L)-Cy5 (715-175-151, Dianova) and anti-rabbit IgG(H + L)-Alexa488 (711-545-152, Dianova).

### In vivo experiments

1205Lu melanoma cells (4 × 10^5^) were injected into the tail vein of Rag2^−/−^γ(c)^−/−^ (6 animals per group). After 15 days, mice were euthanized and lungs were harvested, cut, embedded in paraffin and stained with H&E). All mice procedures were performed in accordance with the relevant ethical guidelines and regulations (authorization number G-18/141). For the zebrafish metastasis model, 9 × 10^5^ MDA-MB-231 cells were suspended in serum-free medium and stained with lipophilic dyes DiO (for CRISPR/Cas9 control) or DiD (for CRISPR/Cas9 *CAD* or *STING*) for 20 min at 37 °C (ThermoFisher Scientific, V22889), prior to injection. Cells were then washed and resuspended in 30 µl of PBS. For zebrafish xenotransplantation, 48 h post-fecundation zebrafish embryos (*Danio rerio*) were dechorionated and anaesthetized with tricaine (Sigma–Aldrich, E10521), and 20 nl of cell suspension (~300 labeled MDA-MB-231 cells) were injected into the perivitelline cavity of each embryo. The embryos were then placed at 30 °C for 48 h and allowed to recover in the presence of N-phenylthiourea (Sigma–Aldrich, P7629) to inhibit melanocyte formation. For imaging and metastasis assessment, zebrafish embryos were anaesthetized with tricaine and imaged using an Axio Observer Zeiss microscope (Zeiss).

### Isolation and analysis of primary intestinal organoids

Small intestine crypts were isolated from wt or CAD-deficient [37] mice and grown as described in the protocol from StemCell Technologies. Synchronization of organoid cultures were performed as described previously (Liccardi et al. 2019). Organoids were further fixed with 4% PFA, permeabilized with PBS-containing 0.5% (v/v) Triton-X and incubated with anti-alpha-tubulin (Sigma, #T9026) and anti-E-cadherin (Cell Signaling, #3195) over-night. Nuclei were stained with DAPI. Chromosome misalignment was assessed using a confocal microscope (Zeiss, LSM-880) with a 40x magnification.

### Micronuclei and chromosome missegregation analysis

For detection of micronuclei by microscopy, 20,000 cells were seeded in an 8-well Ibidi chamber (Ibidi, Gräfelfing, Germany) and incubated for 24 h. Cells were fixed with 4% PFA, washed and stained with DAPI. Images (40x magnification) were taken using a confocal microscope (Zeiss, LSM-880) by a person blinded as to the conditions. For the detection of extranuclear chromosomal fragments by flow cytometry, 20,000 cells in 200 µl of medium were seeded in a 48-well plate and incubated at 37 °C for 72 h. The staining was performed according to the protocol In Vitro Microflow (Litron Laboratories, New York). A FACSCanto II flow cytometer was used to acquire the results using FACSDiva Software and the template In Vitro Microflow by Litron Laboratories for FACSDiva v.6.1.1. FlowJo VX was used for analysis and the gating was performed according to the manufacturer’s protocol. For chromosome missegregation experiments, 30,000 cells were seeded in an Ibidi chamber and incubated for 24 h. Cells were fixed with 4% PFA, washed and stained with anti-alpha-tubulin (Sigma, #T9026) and DAPI. Images were taken under blinded conditions using a confocal microscope (Zeiss, LSM-880). For the detection of cGAS at micronuclei, fixed cells were permeabilized with 0.2% Triton-X, followed by incubation with a cGAS antibody (Cell Signaling, #79978).

### CAD-activation in HaCaT cells

To activate CAD in HaCaT keratinocytes, cells with an auxin-degradable iCAD-mAID-GFP construct were generated as follows: in a first step, we generated iCAD knock-out HaCaT cells using the lentiCRISPR v2 system. Guide RNA for knock-out of human iCAD was obtained from the Brunello database targeting exon#4 of human iCAD (gRNA: ACAGGTGCTTGACCAAAGAG). HaCaT cells were selected with 2 µg/ml puromycin (Invivogen) for 5 days. ICAD-deficient cells were transduced with a lentiviral vector expressing the transport inhibitor response 1-like protein (Tir1 auxin receptor, from *Oryza sativa*) in the hygromycin-selectable vector pFU-G147EV16_PGK_Hygro_W. The cells were selected for 7 days using 800 µg/ml hygromycin. Finally, cells were reconstituted with human iCAD (mutated in the PAM sequence located in exon#4) fused to the minimal Auxin-Induced Degron peptide (IAA17, miniAID (aa65-132) [38, 39] in a lentiviral plasmid (pEF1α-GW-h-iCADMutE4-mAID-GFP-puro). Cells expressing GFP were sorted using flow cytometry.

### Assay for anchorage-independent growth

Cells were seeded in triplicates per experiment. A 2% agarose solution was boiled to 95 °C and was mixed with medium to reach a 0.6% basal-layer agarose solution. The cells were mixed with media and agarose solution to 0.3% agarose in 6-well plates. The plates were incubated at 37 °C and 100 µl of cell culture medium were added dropwise to each well twice per week. After 2–3 weeks of incubation colonies were stained using 0.005% crystal violet solution. The plates were photographed and the stained colonies were counted using ImageJ.

### Invasion assay

Invasion was either tested with a commercial kit or with Matrigel-coated membranes. The CytoSelect 24-Well Cell Invasion Assay (Cell Biolabs, CBA-110) was used, according to the manufacturer’s protocol. Briefly, 5 × 10^5^ cells were resuspended in 1 ml of serum-free medium. After rehydration of the membranes in serum-free medium, the medium was removed from the inserts and 300 µl of the cell suspension were transferred to the inserts. The outside of the insert was filled with 500 µl medium containing 10% serum. After 24 h incubation at 37 °C, the medium was removed and remaining cells on the inside of the inserts were removed using wet cotton swabs. The membranes were stained for 10 min using the provided staining solution, washed and dried. Ten micrographs of each membrane were taken and the number of cells per high power field was counted using ImageJ. Alternatively, stained membranes were incubated for 10 min. with extraction buffer, and the lysate were measured using a microtiter plate reader with an OD of 560 nm. For cell invasion assay using a Matrigel-coated membrane, we used the protocol from Corning as described [40] with modifications. Briefly, individual permeable supports (Corning Cat. No. 353097) were inserted in Falcon cell culture permeable support companion plates (Corning Cat. No. 353504), then coated with 250 µg/ml Corning Matrigel basement membrane matrix (Cat. No. 354234) for 2 h. Cells were then seeded at a density of 5 × 10^4^ cells per condition and allowed to attach overnight. After treatment, cells were left for invasion for 24 h. Non-invasive cells were removed using a wet cotton swap and membranes were fixed with absolute methanol, followed by staining with 1% crystal violet. After drying, five representative images were taken for each permeable support at a magnification of 10x. The cells were counted manually using ImageJ. We changed to this established assay in the course of the project due to cost of the commercial product.

### Migration assay

Cells (5 × 10^5^) were seeded in a 6-well plate. When the cells had reached confluency, a 200 ml pipette tip was used to scratch the monolayer. Wells were washed with PBS three times to remove non-adherent cells. Medium containing 2% serum was added, and photos were taken immediately and twice per day in the same area of each well until the scratches were closed using an Axiovert 40 C microscope. The area of the scratches was quantified using ImageJ and were normalized to the area of the control cell line at 0 h.

### Cell death assays

Per well, 10^5^ cells were seeded in 6-well plates in 2 ml medium each. After 24 h, cells were trypsinised and washed with PBS. Cells were resuspended in 100 µl of AnnexinV Binding Buffer (BD Pharmingen) containing 5 µl of AnnexinV-FITC (eBioscience) and incubated for 15 min on ice in the dark. After washing with PBS, 100 µl of AnnexinV Binding Buffer containing 2.5 µl of 7-AAD (BD Pharmingen) was added and the samples were incubated for 10 min at room temperature. FACSCanto II with FACSDiva software was used for flow cytometry and FlowJo VX was used for data analysis.

### RNA-seq and data analysis

Library preparation for bulk 3’-sequencing of poly(A)-RNA was done as described previously [41]. Briefly, barcoded cDNA of each sample was generated with a Maxima RT polymerase (Thermo Fisher) using oligo-dT primer containing barcodes, unique molecular identifiers (UMIs) and an adapter. 5’ ends of the cDNAs were extended by a template switch oligo (TSO) and after pooling of all samples full-length cDNA was amplified with primers binding to the TSO-site and the adapter. cDNA was fragmented with the Nextera XT kit (Illumina) and 3’-end-fragments finally amplified using primers with Illumina P5 and P7 overhangs. The published protocol [41] was modified in that the P5 and P7 sites were exchanged to allow sequencing of the cDNA in read1 and barcodes and UMIs in read2 to achieve a better cluster recognition. The library was sequenced on a NextSeq 500 (Illumina) with 75 cycles for the cDNA in read1 and 16 cycles for the barcodes and UMIs in read2. Data were processed using the published Drop-seq pipeline (v1.0) to generate sample- and gene-wise UMI tables [42]. Reference genome (GRCh38) was used for alignment.

After excluding genes with non- or low expression levels, 21,827 genes were considered for further analysis of MDA-MB-231 and HeLa cell lines. The PCA plots were generated for each cell line based on the regularized logarithm (rLog) transformation of the counts [43]. Limma [44] was used to identify Differentially Expressed Genes (DEGs) of each cell line under given genotypes (i.e., CAD vs Ctrl and STING vs Ctrl). In order to prepare data before using voom function [44], norm factors were calculated using Trimmed Mean of M-values (TMM) [45]. The numbers of identified DEGs (p-value < 0.05) in the comparison of CAD vs Ctrl are 1612 and 1755 for MDA-MB-231 and HeLa cell lines. In the comparison between STING vs Ctrl, 2313 and 1589 number of DEGs were identified for MDA-MB-231 and HeLa, respectively. The intersections of up- and downregulated DEGs for different genotypes and cell lines are illustrated using Venn diagrams.

For functional enrichment analysis of the identified DEGs, Fisher’s exact test [46] was separately applied to the downregulated genes of each cell line and genotype. We further applied Fisher’s exact test to the overlapping downregulated genes across two cell lines associated with each of the genotypes. For functional analysis, the stats R package was employed using a relevant set of background genes and the consensus term collection. The repeated terms of this collection were excluded using our in house pipeline. For the analysis of the cell lines, the 21,827 genes with higher expression levels were taken as the background. For the overlapping gene sets, the combination of the top DEGs in both cell lines (3254 genes in CAD deficient and 3726 in STING deficient cell lines) was taken as the background. After functional enrichment analysis, consensus terms with a *p* < 0.05 were retained. To provide enrichment analysis of 693 expressed EMT genes [47], the R package fgsea (Sergushichev, A *An algorithm for fast preranked gene set enrichment analysis using cumulative statistic calculation*. bioRxiv. doi: 10.1101/060012 (2016)) was used. For the heatmaps, the *z* score of the log transformation of the gene expression of the top DEGS was used. In the case of the heatmaps of the EMT genes, the set of top 50 EMT genes which are deregulated in CAD deficient HeLa cell line and STING-deficient MDA-MD-231 cell line were used, accordingly. RNA-seq data were deposited at the GEO database (accession number GSE155493).

### Survival analysis

For each of the first and second signatures, down regulated genes (adjusted *p* < 0.01) in CAD deficient HeLa and MDA-MB-231 cell lines were used (Table S1, S2). After identifying three CAD deficient gene signatures, TCGA data collection was used for survival analysis. A total of 329 patients for whom clinical data together with tumor and normal pairs of RNAseq were available, were included in this analysis. Cancer entities include colon adenocarcinoma, lung adenocarcinoma, prostate adenocarcinoma, stomach adenocarcinoma, uterine corpus endometrial carcinoma, pancreas adenocarcinoma, lung squamous cell carcinoma, head and neck squamous cell carcinoma, esophageal carcinoma, cervical squamous cell carcinoma and endocervical adenocarcinoma, breast invasive carcinoma, and bladder urothelial carcinoma. For each patient, the level of log2-fold change (log2FC) was computed for each gene comparing normal and tumor tissue. The value of log2FC determines if a gene of the CAD-deficient signature is up or downregulated due to the development of tumor disease, and how strong it is deregulated. The survival package in R was used to fit the multivariate Cox proportional hazards model (CoxPH) for each signature. The logarithm of hazard ratio of each variable (beta coefficients of the model) and corresponding p-value is provided in Tables S1, S2.

The association of the CAD-signature with patient survival was evaluated using the Kaplan–Meier plotter tool [48] and the logrank (Mantel Cox) test with 95% Confidence Interval. We analyzed 12 cancer patient cohorts from the The Cancer Genome Atlas (TCGA) (bladder/urothelial carcinoma, breast invasive carcinoma, cervical squamous cell carcinoma and endocervical adenocarcinoma, cholangiocarcinoma, colon adenocarcinoma, esophageal carcinoma, head and neck squamous cell carcinoma, lung adenocarcinoma, lung squamous cell carcinoma, rectum adenocarcinoma, stomach adenocarcinoma and uterine corpus endometrial carcinoma). Patients were stratified into groups based on the highest and lowest expressing quartile. Three separate studies of gene expression profiles in breast cancer [49], gastric cancer [50] or non-small cell lung cancer [51] were analyzed separately. Patients were assigned into high and low expression groups using the auto-selection for best cutoff option between the lower and upper quartiles and only optimal probe set for each gene was selected using the JetSet best probe set option. The 19-gene CAD-signature assessed for association with overall survival or distant metastasis-free survival was further validated using Gene Expression Profiling Interactive Analysis (GEPIA) [52].

### Statistical analyses

Statistical analyses were performed using Prism 8 (GraphPad) software. Parametric *t* test (with Welch’s correction) was used for normally distributed datasets. Survival curves were compared using log-rank Mantel-Cox test. Data were considered significant when *P* ≤ 0.05, with *P ≤ 0.05, **P ≤ 0.01, ***P ≤ 0.001 or ****P ≤ 0.0001.

## Supplementary information

Supplementary Figures and Tables

Original confocal pictures

Original blot 2

Original blot

aj-checklist

## Data Availability

RNA-seq. data have been deposited RNA-seq data at the GEO database (accession number GSE155493). The datasets generated during and/or analyzed during the current study are available from the corresponding author.
