# Supplementary material for: Spontaneous activity of the mitochondrial apoptosis pathway drives chromosomal defects, the appearance of micronuclei and cancer metastasis through the Caspase-Activated DNAse
Source: Cell Death Dis. 2022 Apr 7;13(4):315. doi: 10.1038/s41419-022-04768-y (PMC8990075; doi:10.1038/s41419-022-04768-y)
Supplement: Supplementary file 1 — Supplementary Figures and Tables [file 41419_2022_4768_MOESM1_ESM.pdf]

Supplementary Fig.1

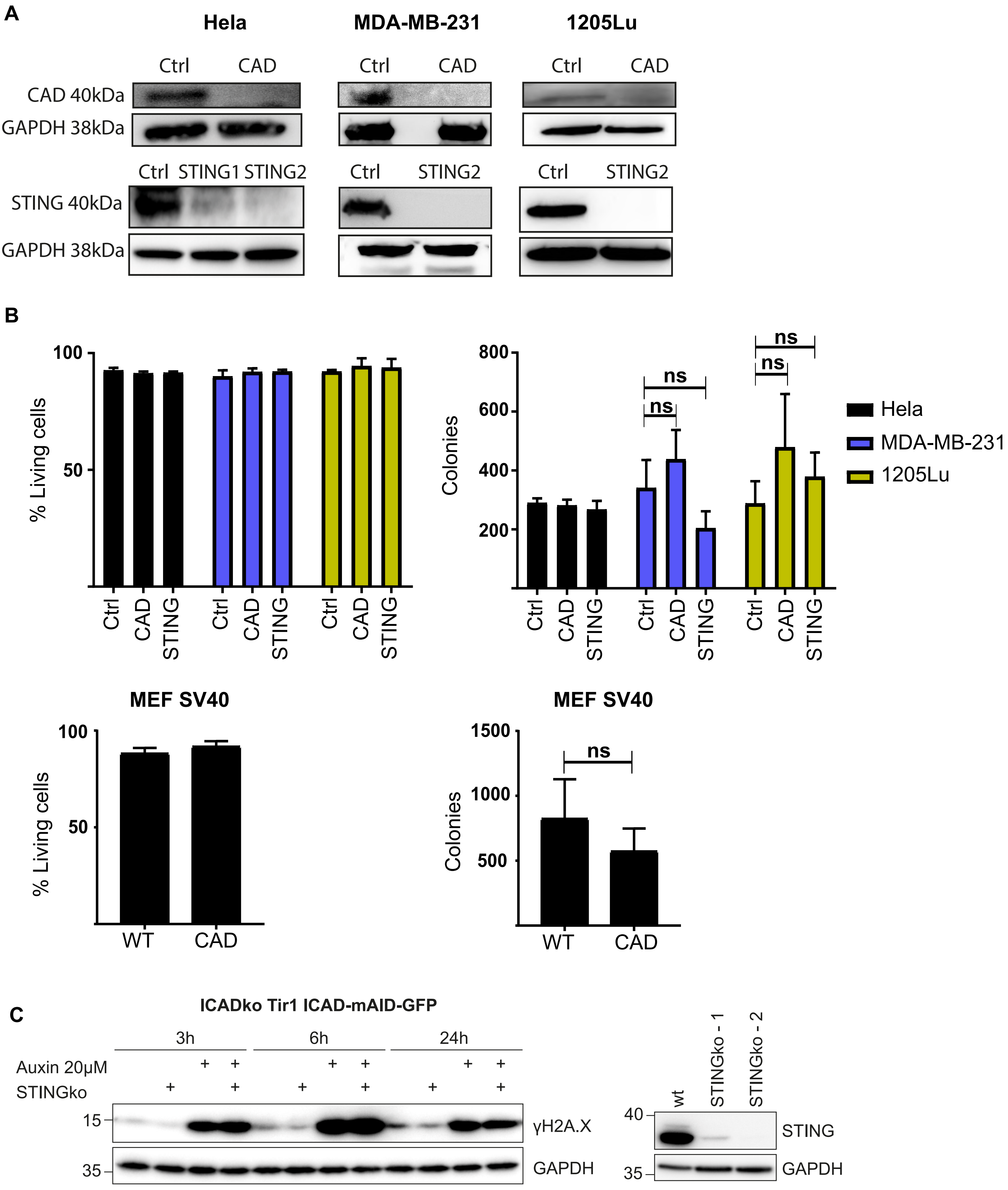

Supplementary Fig.2

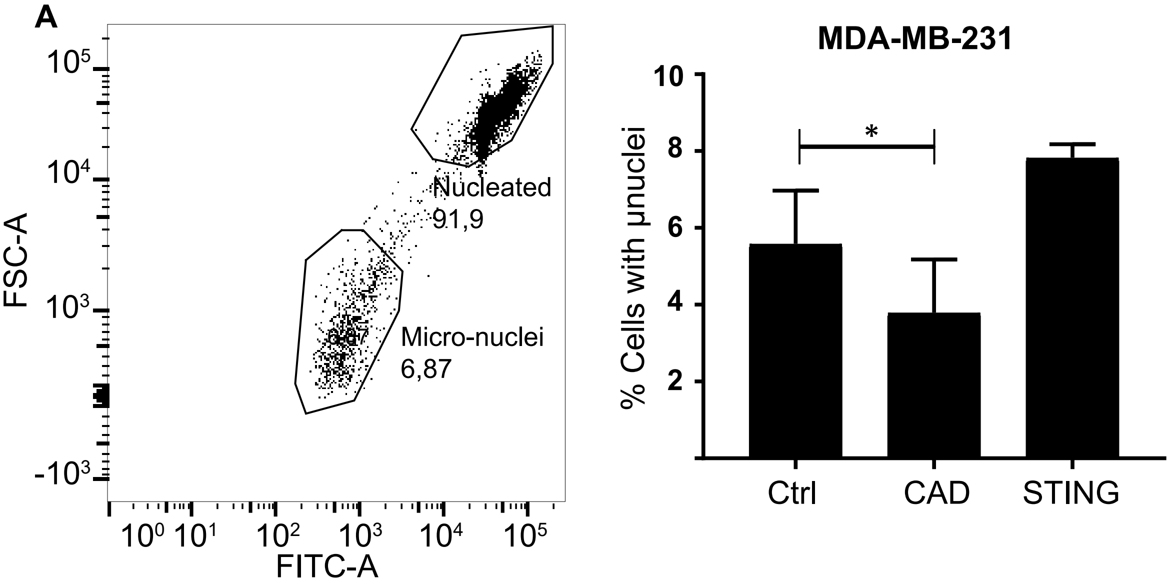

Supplementary Fig.3

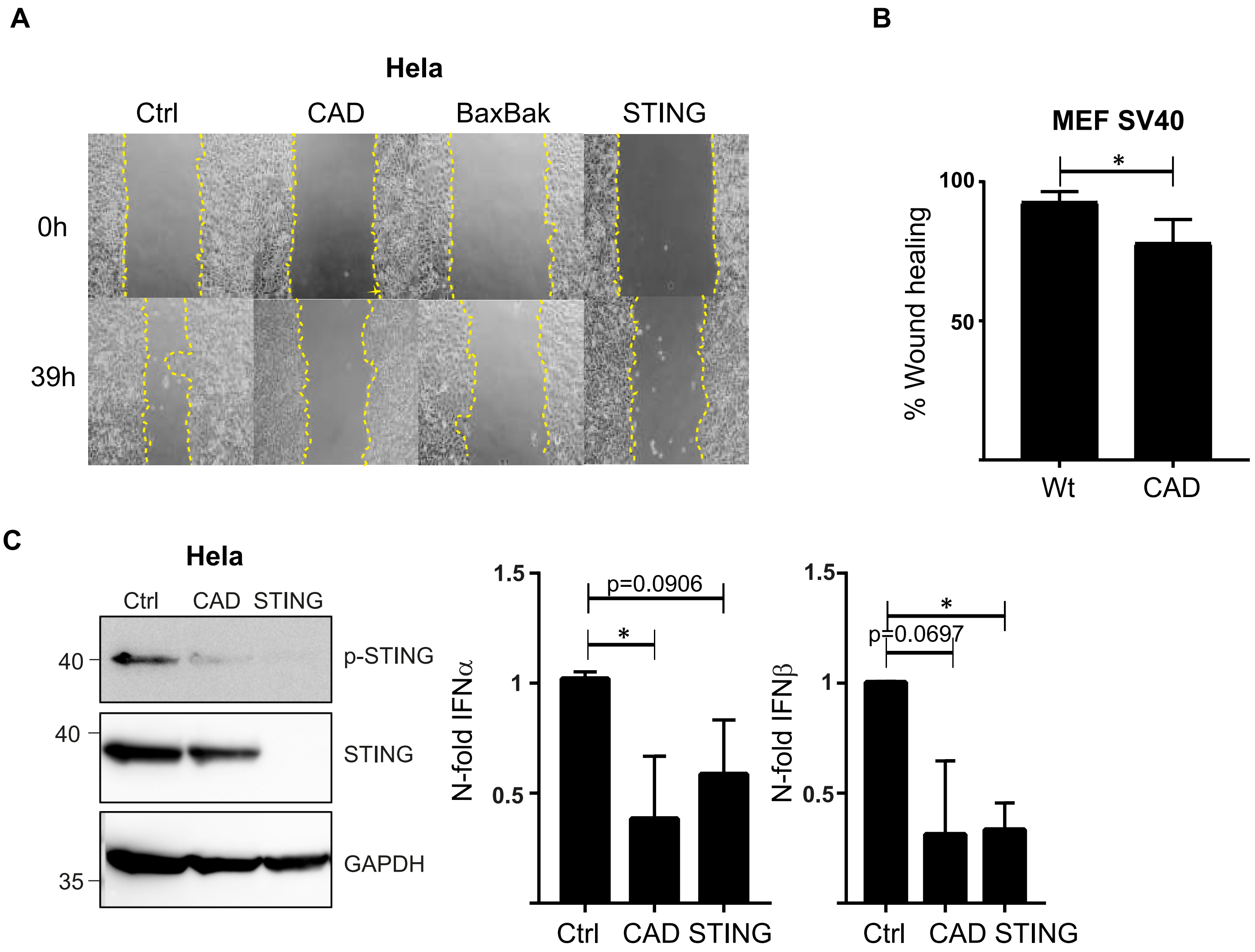

Supplementary Fig.4

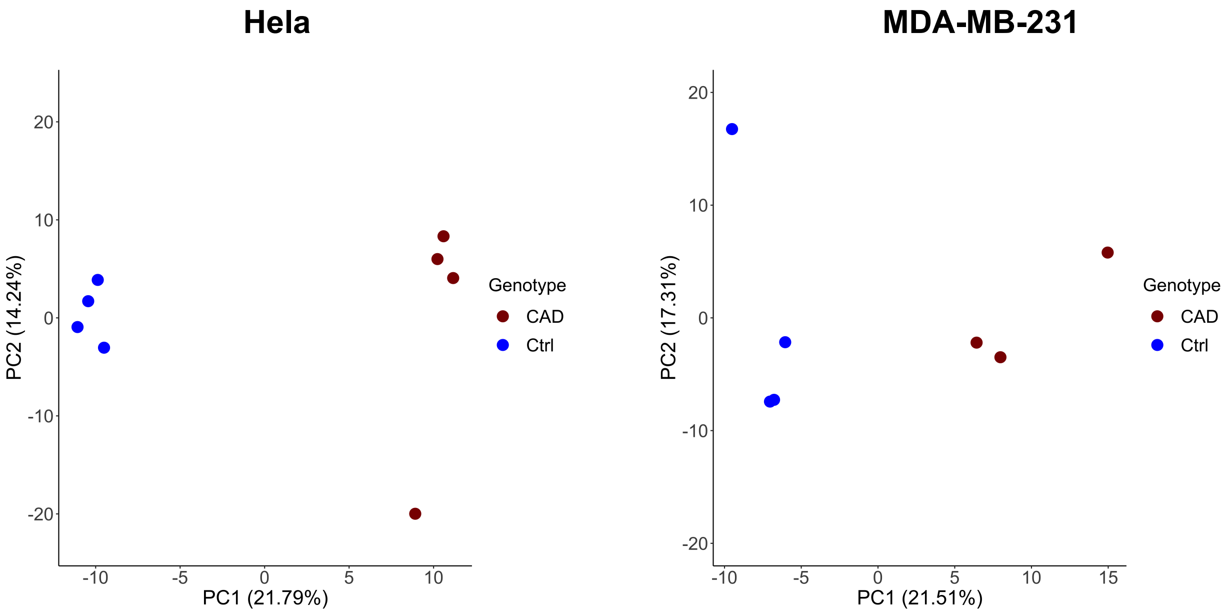

Supplementary Fig.5

A

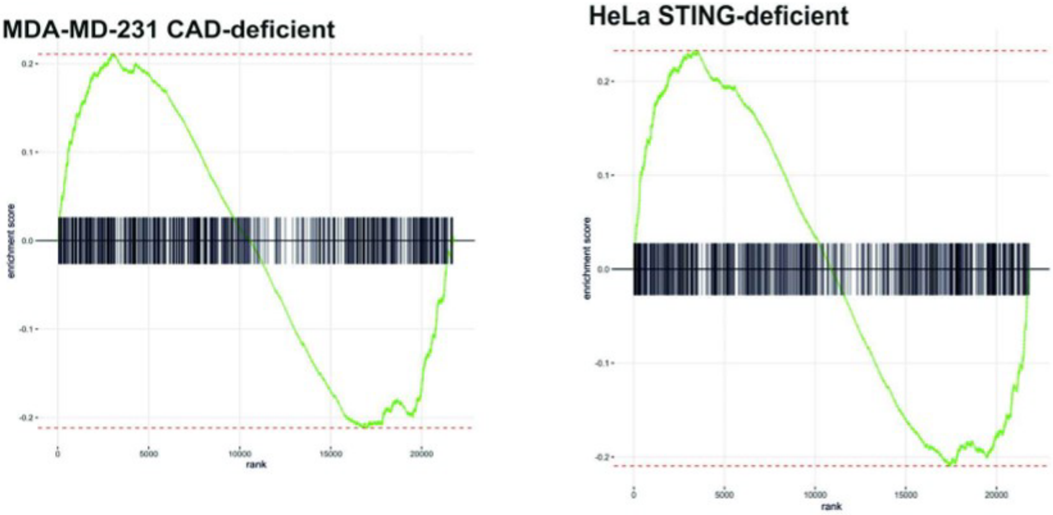

B

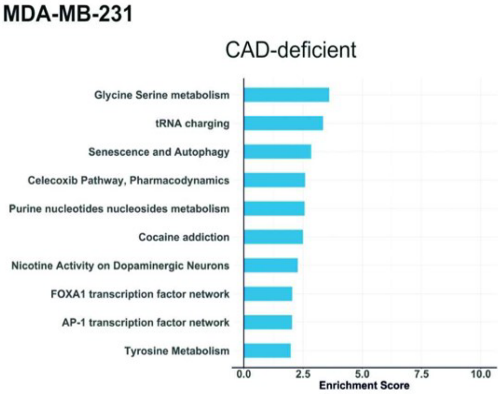

Supplementary Fig.6

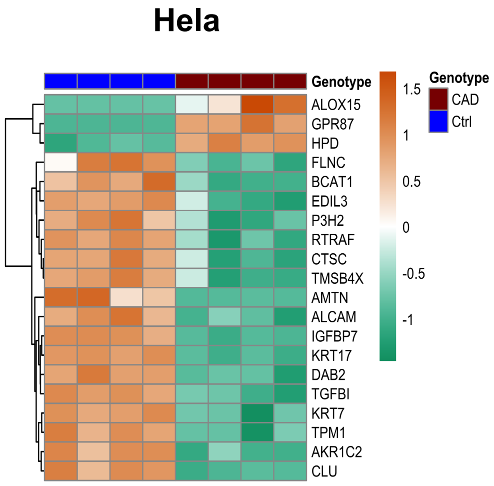

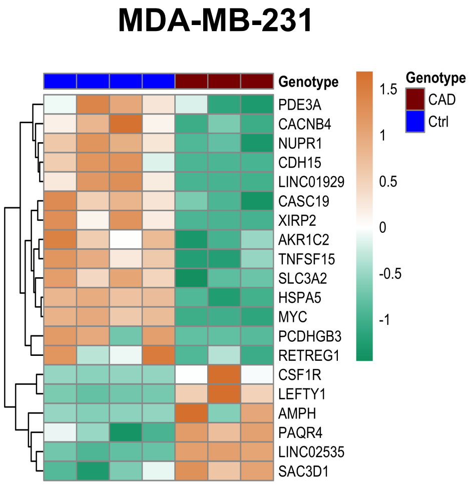

Supplementary Fig.7

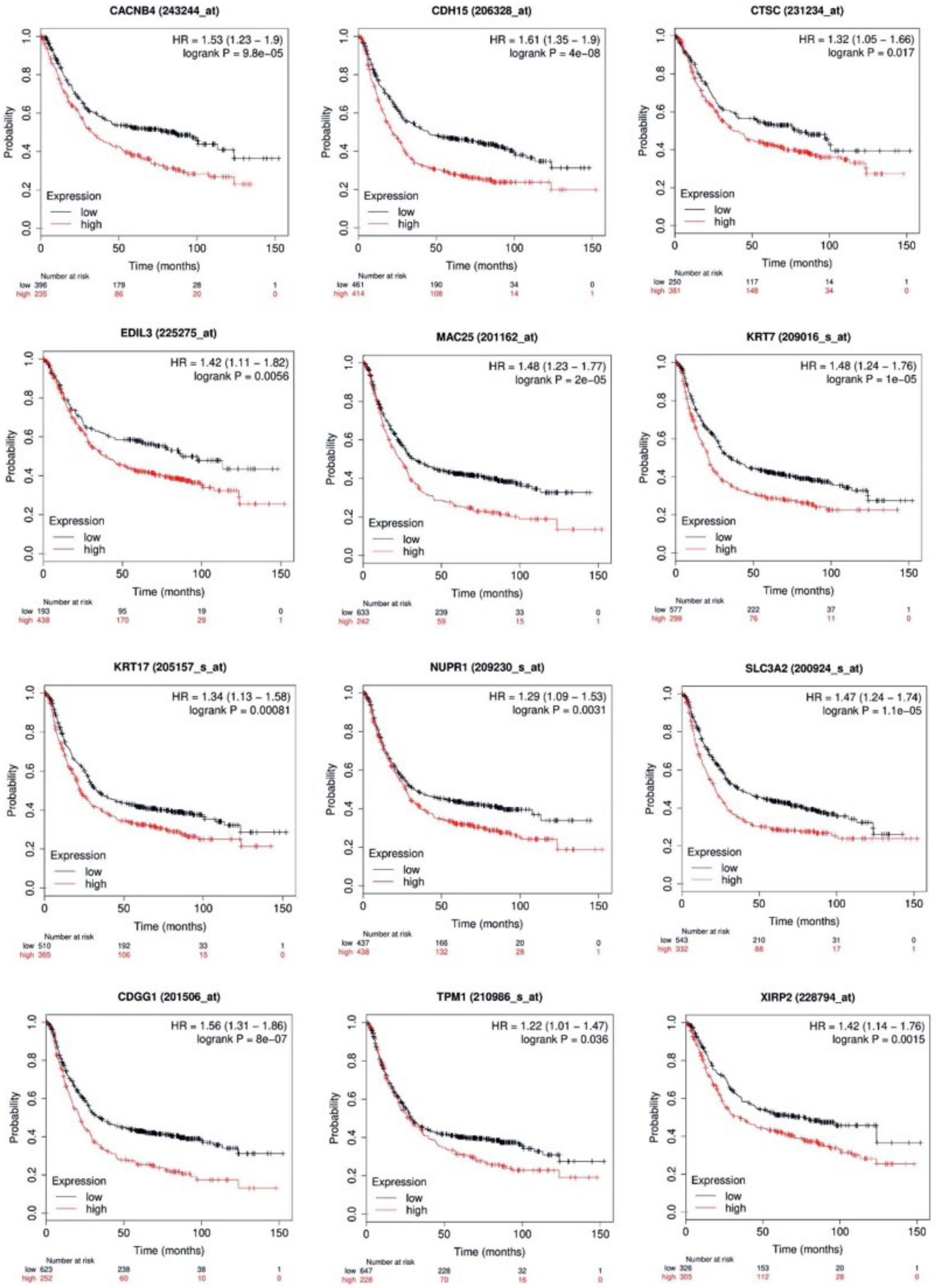

Table S1

COXPH results for significantly downregulated genes (adjusted pValue < 0.01) in CAD-deficient HeLa cells

For each gene the estimation of log Hazard Ratio (log HR or parameter beta) together with related confidence interval and pValue is given.

The significance of each gene is defined based on the following criteria: '***' pValue < 0.001; '**' pValue < 0.01; '*' pValue < 0.05

term	estimate	conf.low	conf.high	pVal	signif.
PI16	-0,13865	-0,24477	-0,03253	0,010442	*
FTH1	0,329754	0,002327	0,65718	0,048394	*
TGFBI	0,204859	-0,01824	0,427955	0,071901	
CLU	0,153275	-0,01657	0,323124	0,076941	
COL4A1	0,298811	-0,0358	0,633422	0,080072	
IGFBP3	0,136202	-0,01651	0,288919	0,080462	
CCND1	-0,15891	-0,3503	0,032481	0,103666	
ITGA11	0,187212	-0,04322	0,417647	0,111309	
LOX	-0,19282	-0,43866	0,053018	0,124226	
LINC02104	0,120503	-0,04071	0,281717	0,142915	
COL5A1	-0,22884	-0,55896	0,101282	0,174259	
RTRAF	-0,3257	-0,82283	0,17143	0,199109	
PYCARD	0,177882	-0,09427	0,45003	0,200166	
PERP	0,144119	-0,07833	0,366573	0,204161	
SPINK6	-0,05143	-0,13292	0,030063	0,216128	
DAB2	-0,17963	-0,47108	0,111824	0,227058	
ALCAM	0,111276	-0,06986	0,292415	0,228581	
ACTB	-0,29281	-0,77603	0,190412	0,234971	
C11orf24	0,288034	-0,20044	0,776511	0,247802	
FLNC	0,080839	-0,09477	0,256449	0,366931	
CTSC	-0,13139	-0,42406	0,161275	0,378901	
AGTRAP	-0,15016	-0,50634	0,20602	0,408635	
TMSB4X	-0,16049	-0,54724	0,226271	0,416051	
AKAP12	0,068163	-0,1001	0,236428	0,427219	
TIMP1	0,103357	-0,16059	0,367306	0,442798	
MYL9	-0,15184	-0,56064	0,256967	0,466632	
BDNF	-0,03857	-0,15318	0,076041	0,509532	
MFAP5	0,041319	-0,0849	0,167541	0,521138	
AMTN	0,029099	-0,06038	0,118576	0,523867	
P3H2	-0,04729	-0,19401	0,099433	0,527577	
PRDX4	0,117846	-0,27848	0,514168	0,560031	
TPM1	-0,0753	-0,33994	0,18934	0,577062	
KRT17	0,024526	-0,07868	0,127727	0,641371	
PRSS23	0,048623	-0,17067	0,267916	0,663871	
GPRC5A	-0,02586	-0,16187	0,110158	0,709444	
CGA	-0,01601	-0,10395	0,071926	0,72115	
TAGLN	-0,06764	-0,51575	0,380466	0,76734	
DCN	0,032028	-0,21051	0,274564	0,79577	
AKR1C2	-0,01135	-0,10107	0,078365	0,804143	
CRYAB	-0,01998	-0,18129	0,141331	0,808206	
KRT7	-0,01151	-0,11176	0,088751	0,822035	

Table S2

COXPH results for significantly downregulated genes (adjusted pValue < 0.01) in CAD-deficient MDA-MB-231 cells

For each gene the estimation of log Hazard Ratio (log HR or parameter beta) together with related confidence interval and pValue is given.

The significance of each gene is defined based on the following criteria: '***' pValue < 0.001; '**' pValue < 0.01; '*' pValue < 0.05

term	estimate	conf.low	conf.high	pVal	signif.
SLC5A3	0,280724	0,088873	0,472575	0,004132	**
CACNB4	-0,13837	-0,25778	-0,01896	0,023138	*
MYC	-0,12971	-0,25134	-0,00808	0,036598	*
PDE3A	-0,10154	-0,20305	-3,7E-05	0,049917	*
CASC19	0,072497	-0,00132	0,146316	0,054248	
SLC3A2	0,241679	-0,01329	0,496647	0,063196	
AKR1C2	0,047635	-0,01197	0,107241	0,117279	
CDH15	0,061545	-0,02825	0,15134	0,179159	
TNFSF15	-0,07082	-0,1972	0,055566	0,272098	
PCDHGB3	0,060496	-0,05906	0,180055	0,321325	
XIRP2	-0,03284	-0,10025	0,034569	0,339636	
NUPR1	-0,0615	-0,19627	0,073267	0,371084	
HSPA5	-0,11045	-0,39431	0,173415	0,445707	
EMX1	-0,00291	-0,06797	0,062158	0,930265	

Table S3

COXPH results for the top 10 downregulated genes in CAD-deficient Hela or MDA-MB-231 cells. AKR1C2 was down-regulated in both CAD-deficient cells lines.
For each gene the estimation of log Hazard Ratio (log HR or parameter beta) together with related confidence interval and pValue is given. Genes downregulated in STING-deficient cells (Hela and/or MDA-MB-231) or unique for CAD-deficient cells are indicated.
The significance of each gene is defined based on the following criteria: '***' pValue < 0.001; '**' pValue < 0.01; '*' pValue < 0.05

term	estimate	conf.low	conf.high	pVal	signif.	Downregulated in STING-deficient Hela and/or MDA (H/M)
CTSC	-0,34552	-0,5666	-0,12444	0,00219	**	M
CACNB4	-0,18235	-0,31156	-0,05314	0,005673	**	unique for CAD-deficient cells
KRT7	0,076855	0,007675	0,146034	0,029451	*	H/M
TGFBI	0,159614	0,004708	0,31452	0,043432	*	H/M
AKR1C2	0,062353	-0,00072	0,125424	0,052668		unique for CAD-deficient cells
SLC3A2	0,263334	-0,01506	0,541724	0,063743		H/M
KRT17	0,048523	-0,01293	0,109979	0,121744		H/M
TNFSF15	-0,10708	-0,25994	0,045773	0,169739		H/M
CASC19	0,049823	-0,02183	0,121475	0,172925		unique for CAD-deficient cells
MYC	-0,0889	-0,22377	0,04598	0,196428		H/M
IGFBP7	-0,13079	-0,33033	0,068744	0,198892		H/M
BCAT1	0,079091	-0,06831	0,226489	0,292948		unique for CAD-deficient cells
TMSB4X	0,141945	-0,13321	0,417104	0,311981		H
NUPR1	-0,08027	-0,24314	0,082606	0,334084		H/M
XIRP2	-0,03718	-0,11443	0,040066	0,345459		H/M
TPM1	0,055979	-0,07638	0,188339	0,407143		H/M
EDIL3	0,043202	-0,08642	0,172827	0,513612		H
HSPA5	0,087884	-0,20348	0,379251	0,554401		H/M
CDH15	-0,02282	-0,11559	0,069948	0,629692		H/M

Supplementary Figure legends

Supplemental figures

Figure S1 *Normal behaviour of CAD- and STING-deficient cells in standard cell culture*

A, Confirmation of the loss of CAD or STING protein by Western blotting**. B**, Cells (HeLa cervical carcinoma cells, MDA-MB-231 breast carcinoma cells, 1205Lu melanoma cells and SV40-transformed MEF cells) deficient for CAD or STING were tested for cell viability by annexin V/7-AAD-staining and flow-cytometry (number of cells negative for both dyes is given, left panels). Cell growth was determined by colony-formation assay on cell culture plates. Data are means/SEM of three independent experiments. Ns, $p>0.05$.

C, HaCaT AID-ICAD-GFP cells were used to delete STING using CRISPR/Cas9 (STING-2 was further used for this study, right blot). Cells were treated with auxin (20 µM) for the times indicated. Western blot shows loss of ICAD and the appearance of a DNA-damage response (γH2AX) over time (left panel). Confirmation of the loss of STING is shown in the right panel.

Figure S2 *Spontaneous CAD-activity drives the appearance of micronuclei*

Micronuclei were detected by flow cytometry. Cells were lysed, stained with SYTOX Green, and micronuclei were detected as shown in the flow cytometry chart (left panel). Percent micronuclei per total events from three independent experiments with MDA-MB-231 cells carrying a non-coding gRNA (ctrl.) or deficient in CAD or STING are shown. Data shown are means/SD. *, $p < 0.05$.

Figure S3 *The mitochondrial apoptosis pathway and CAD determine growth behavior of tumor cells* in vitro

A Representative image of the scratch assay with HeLa cells, 0 and 39 h after scratching (data are part of Fig. 3A).

B Quantitation of phase-contrast images of the scratch assay using SV40-transformed MEF cells. Data shown are means/SD of three independent experiments. *, $p < 0.05$.

C Western Blot for phospho-STING and STING in Ctrl, CAD- and STING-deficient Hela cells (left panel). GAPDH was used as a loading control. Right panel: RT-PCR analysis of type I interferon genes. Data shown are means/SD of four independent experiments. *, $p < 0.05$.

Figure S4 *Clustering of individual genotypes*

PCA plots show distinctive clustering of Hela and MDA-MB-231 cells across different genotypes using RNA-expression analysis.

Figure S5 *CAD drives a cell-specific gene expression profile*

A Enrichment analysis of EMT-related genes in CAD-deficient MDA-MB-231 (left) and STING-deficient Hela (right) cell lines. **B** Functional enrichment analysis in CAD-deficient MDA-MB231 cells using Fisher's exact test. The top-10 negatively enriched consensus terms are shown in the bar plots. The enrichment score presents the corresponding log10 p-value.

Figure S6 *Heat maps of genes deregulated in CAD deficiency*

Top 20 DEGs in CAD-deficient HeLa (left panel) and MDA-MB-231 (right panel) cells.

Figure S7 *Association of individual genes of the CAD-dependent signature with survival in cancer patients*.

The association of the tumor tissue-expression of individual genes from the CAD-dependent gene signature with survival in a cohort of gastric cancer patients. The 12 genes whose expression was significantly associated with survival are shown. Patients were divided into

high and low expression groups for each gene according to the best cutoff between the lower and upper quartiles (CACNB4, CTSC, EDIL3, XIRP2 n=631, other genes n=875).

Table S1

CoxPH results for significantly downregulated genes (adjusted pValue < 0.01) in CAD-deficient HeLa cells

Table S2

CoxPH results for significantly downregulated genes (adjusted pValue < 0.01) in CAD-deficient MDA-MB-231 cells

Table S3

CoxPH results for the top 10 most significantly downregulated genes in CAD deficient Hela or MDA-MB-231 cells. One gene (AKR1C2) was downregulated in both cell lines, the other ones exclusively in CAD deficient Hela or MDA-MB-231 cells
