# Supplementary figures and images for: Spontaneous activity of the mitochondrial apoptosis pathway drives chromosomal defects, the appearance of micronuclei and cancer metastasis through the Caspase-Activated DNAse
Source: Cell Death Dis. 2022 Apr 7;13(4):315. doi: 10.1038/s41419-022-04768-y (PMC8990075; doi:10.1038/s41419-022-04768-y)

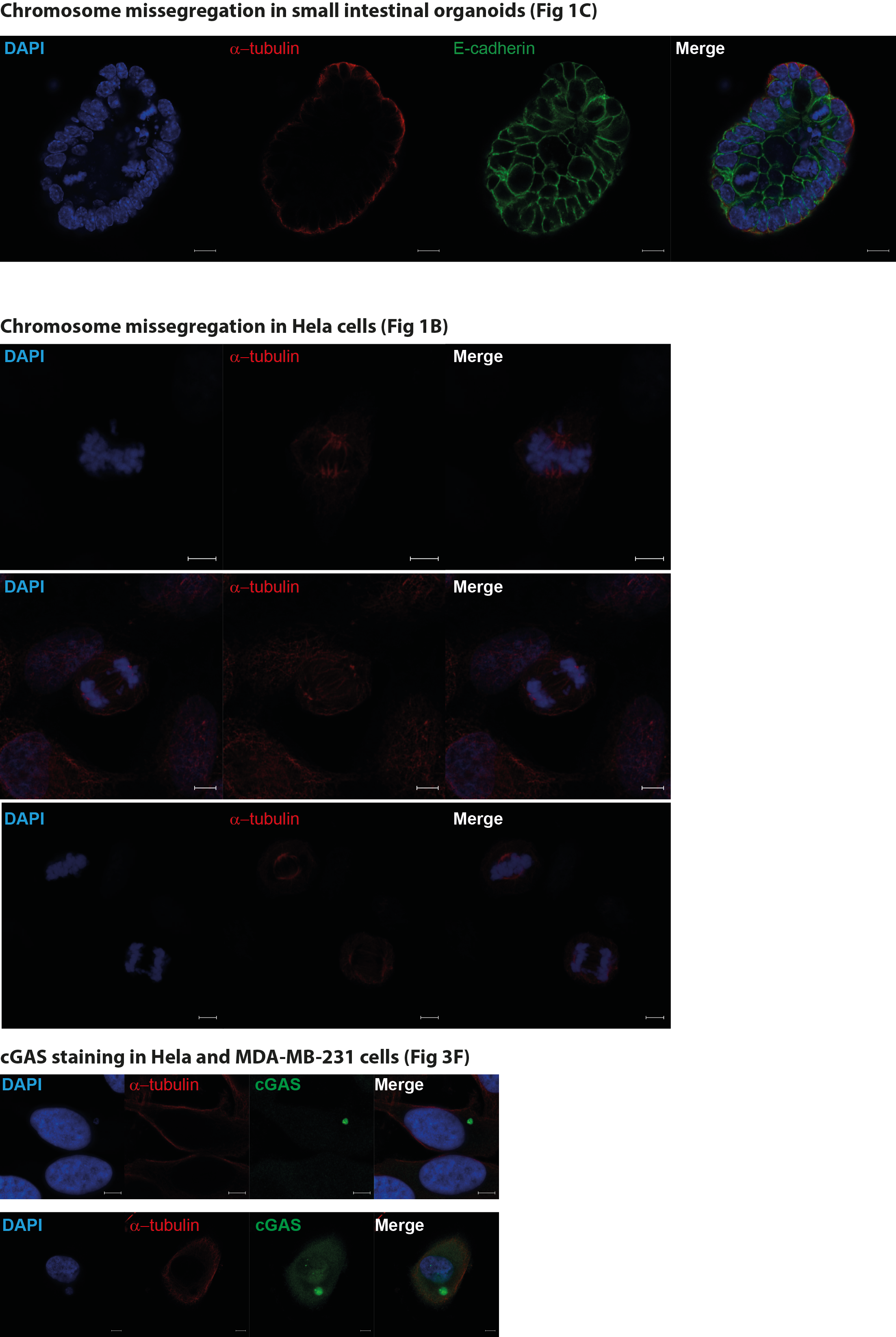

Supplement: Supplementary file 2 — Original confocal pictures [file 41419_2022_4768_MOESM2_ESM.png]

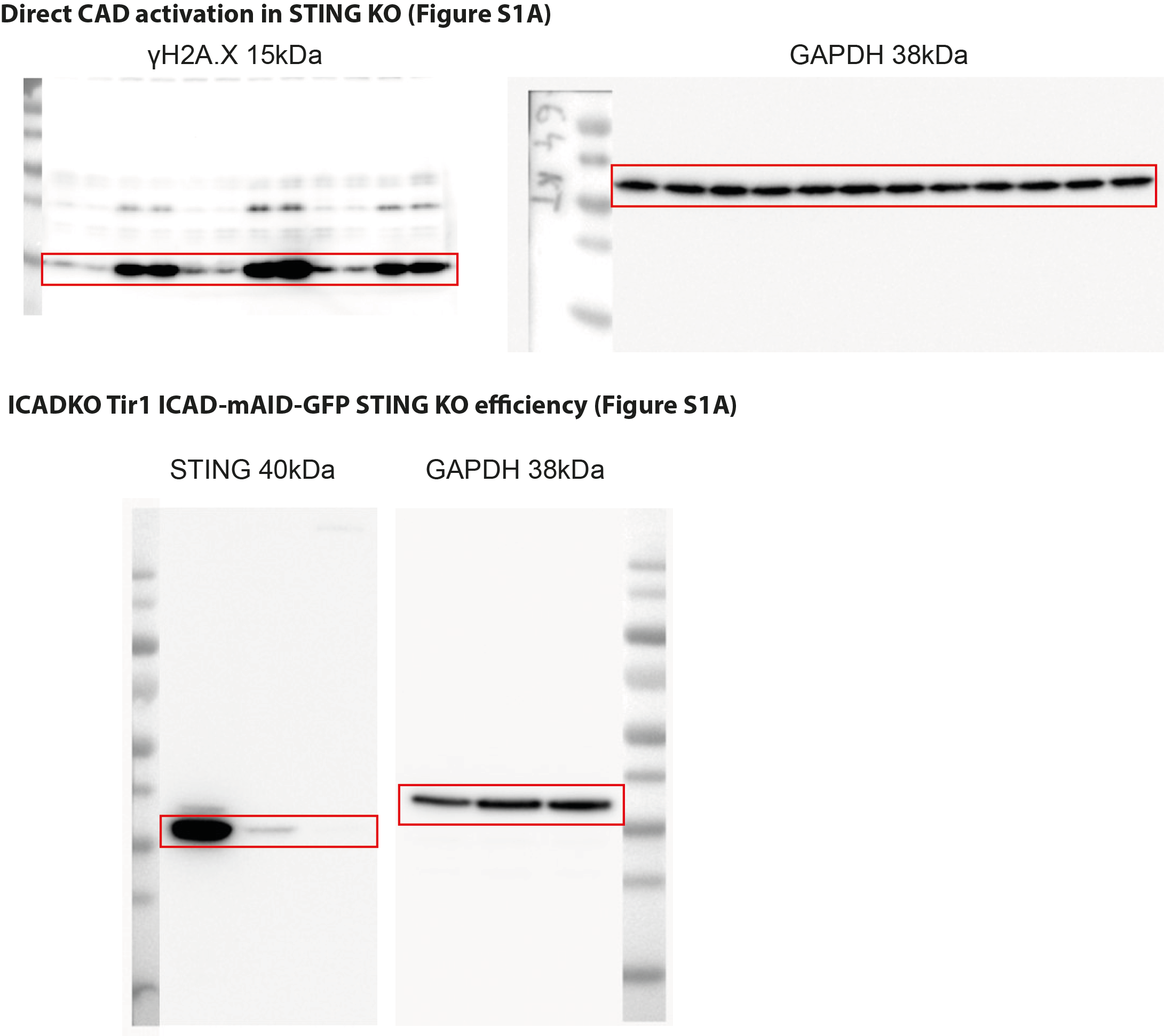

Supplement: Supplementary file 3 — Original blot 2 [file 41419_2022_4768_MOESM3_ESM.png]

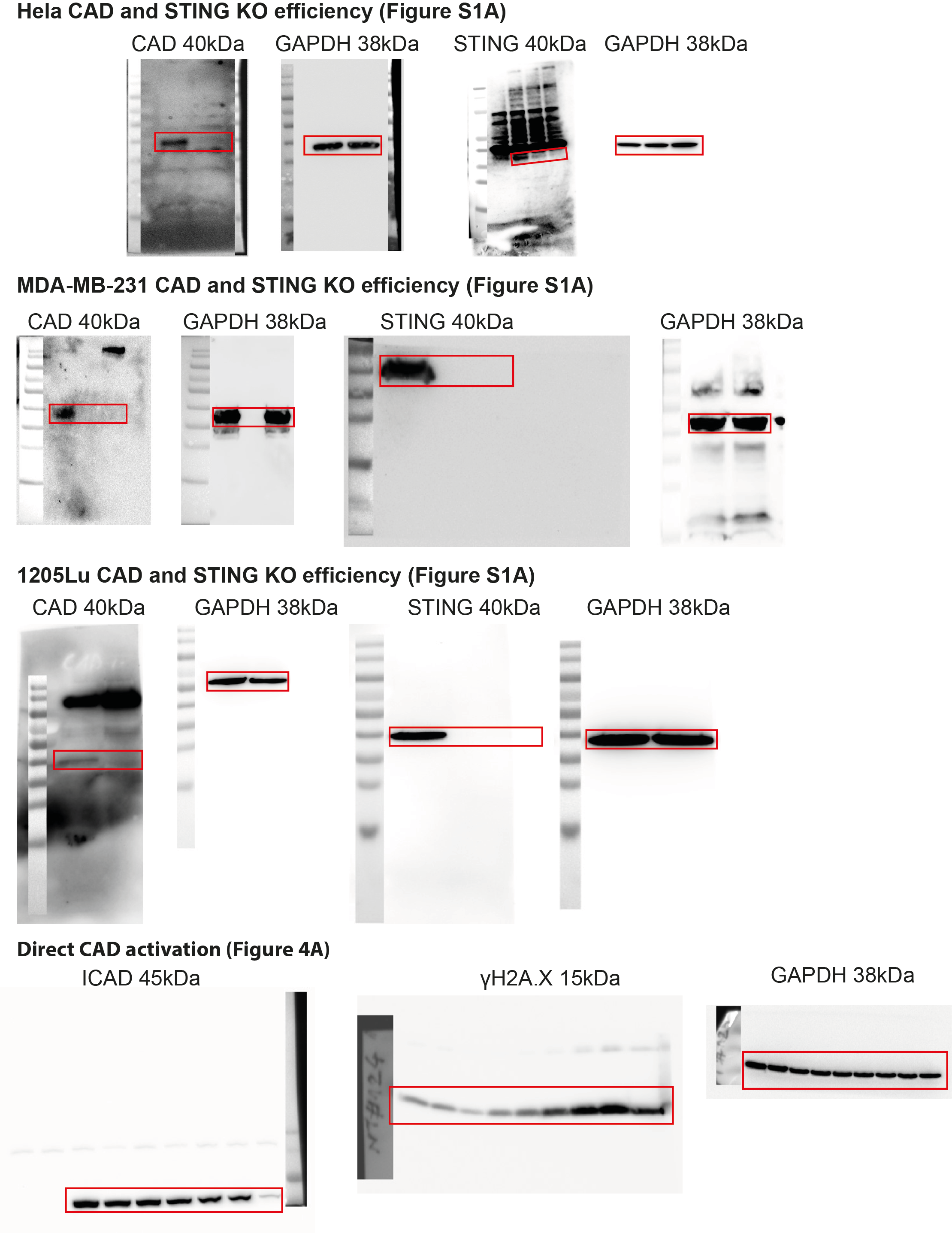

Supplement: Supplementary file 4 — Original blot [file 41419_2022_4768_MOESM4_ESM.png]
